# Critical assessment of anti-amyloid-β monoclonal antibodies effects in Alzheimer’s disease: a systematic review and meta-analysis highlighting target engagement and clinical meaningfulness

**DOI:** 10.1038/s41598-024-75204-8

**Published:** 2024-10-28

**Authors:** Konstantinos I. Avgerinos, Apostolos Manolopoulos, Luigi Ferrucci, Dimitrios Kapogiannis

**Affiliations:** 1 Department of Neurology, Wayne State University, Detroit Medical Center, University Health Center, 8th floor, 4201 St. Antoine, Detroit, MI 48201 USA; 2 Intramural Research Program, Laboratory of Clinical Investigation, National Institute on Aging, NIH, Baltimore, MD 21224 USA; 3 Intramural Research Program, Longitudinal Studies Section, National Institute on Aging, NIH, 251 Bayview Blvd, Ste 8C228, Baltimore, MD 21224 USA

**Keywords:** Drug discovery, Neuroscience, Neurology

## Abstract

Despite most monoclonal antibodies against Aβ in Alzheimer’s failed to demonstrate efficacy, the newest antibodies showed statistically significant clinical effects. We conducted a systematic review and meta-analysis to assess the efficacy, target engagement, and safety of anti-Aβ antibodies in sporadic AD including phase III RCTs published up to November 28, 2023. Antibodies as a drug class, attenuated worsening on the clinical scales CDR-SB and ADAS-Cog by very small effect sizes and reduced amyloid on PET by a very large effect size. Reduction of amyloid on PET was moderately correlated with CDR-SB and ADAS-Cog reductions. However, antibodies increased risk of ARIA-E and ARIA-H by a very large and moderate effect size, respectively. In subgroup analyses by individual drug, Donanemab and Lecanemab induced the largest benefits. In subgroup analyses by binding affinity, antibodies without binding to monomers were associated with the most favorable effects. Despite statistical significance for improvement on clinical measures, antibody effects were below the threshold of clinically meaningful change during the period they were studied. However, the newest antibodies demonstrably interfere with the underlying ΑD pathophysiology and therefore their benefit could be cumulative over time leading to larger clinical effects in subsequent years. PROSPERO registration no. CRD42022381334.

**Supplementary Information:**

The online version contains supplementary material available at 10.1038/s41598-024-75204-8.

## Introduction

The amyloid hypothesis postulates that deposition of amyloid-beta (Aβ) pathology in the brain drives tau pathology, neurodegeneration, and ultimately cognitive decline in Alzheimer’s disease (AD)^1,2^. Over the last decades, based on the amyloid hypothesis, drugs aiming to either decrease production of Aβ (e.g., beta and gamma-secretase inhibitors) or increase its clearance (e.g., active and passive immunization) have been developed. Among all anti-amyloid approaches, passive immunization with monoclonal antibodies (mAbs) against Aβ has risen as the most promising strategy, given their mechanistic selectivity and tolerability^3^. Although the vast majority of anti-Aβ mAbs such as Bapineuzumab, Solanezumab, Crenezumab, and Gantenerumab failed to demonstrate efficacy, newer mAbs including Aducanumab, Lecanemab, and Donanemab induced statistically significant benefits on several clinical measures leading to their FDA approval. However, their effects were of small effect sizes^4–6^, thereby generating controversy over their clinical significance^7,8^. For Aducanumab, additional concerns have been expressed because results from one trial were not replicated in a second similarly-designed trial^9^.

Differences in effects between individual mAbs, inconsistencies in effects of individual mAbs across trials, and controversies over the clinical importance of statistically significant effects reveal the current uncertainty on the efficacy of anti-Aβ mAbs in AD. We conducted a systematic review and meta-analysis with the aim to synthesize and critically appraise all existing evidence on the efficacy, target engagement, and safety of anti-Aβ mAbs as a class and at the level of individual drug in sporadic AD. We additionally investigated whether the degree of amyloid reduction was associated with clinical efficacy. Furthermore, Aβ can be found in various forms, including monomers, oligomers, protofibrils, fibrils, and plaques, and each mAb binds to specific combinations of them. Some evidence suggests that Aβ monomers may play a protective role in AD^10^ and, as a result, mAbs binding to monomers may lead to less favorable clinical outcomes. Therefore, we tested whether strength of binding for monomers was associated with clinical outcomes.

## Methods

The protocol of this systematic review and meta-analysis has been registered in PROSPERO (registration no. CRD42022381334). We report our methods and results in accordance with the Preferred Reporting Items for Systematic Reviews and Meta-analyses (PRISMA) statement (Table S1)^11^.

### Data sources

We searched MEDLINE, Embase, and Cochrane Central Register of Controlled Trials (CENTRAL) from inception to November 28, 2023. We used relevant free-text and controlled vocabulary terms without language or publication status restrictions. The detailed search strategy is available in Table S2. We also searched clinical trial registries, including ClinicalTrials.gov, European Union Drug Regulating Authorities Clinical Trials (EudraCT) Database, and the WHO International Clinical Trials Registry Platform. Finally, we manually searched for additional studies in the proceedings of Clinical Trials on Alzheimer’s Disease (CTAD), the Alzheimer’s & Parkinson’s Diseases (AD/PD), and the Alzheimer’s Association International (AAIC) conferences, and the references of retrieved articles and pertinent reviews.

### Study selection

We synthesized results of phase III randomized controlled trials (RCTs) of mAbs against any Aβ species, irrespective of dosage regimen, in adults with sporadic AD. We selected phase III RCTs because unlike early phase (I, II) RCTs, they employed more rigorous and largely consistent designs and comparable mAb doses and treatment durations allowing for synthesis of more homogeneous data. Case reports, case series, observational studies, single-arm trials, non-human studies, and studies on autosomal dominant AD were excluded.

Records retrieved from electronic databases and grey literature sources were imported into a reference management software. After deduplication, the remaining records were exported to an online software (Covidence; Veritas Health Innovation Ltd, Melbourne, Australia) for screening. Two reviewers independently (KA, AM) screened all records at title and abstract level and subsequently assessed the full texts of potentially eligible studies with reasons for exclusions being recorded. Any disagreements were resolved by consensus between the two reviewers. The final set of studies was included in the systematic review (qualitative portion of our study), with only studies providing numerical results for mAb comparison to placebo being included in meta-analyses (quantitative portion of our study).

### Data extraction

Two reviewers (KA, AM) independently extracted data for study characteristics, participants’ demographics and baseline characteristics, and pre-specified outcomes from eligible studies using a pre-designed extraction form; any discrepancies were arbitrated by a senior reviewer (DK). Our primary outcome was the Clinical Dementia Rating scale-Sum of Boxes (CDR-SB). Secondary outcomes included the AD Assessment Scale-Cognitive Subscale (ADAS-Cog), Mini Mental State Examination (MMSE), and AD Cooperative Study-Activities of Daily Living (ADCS-ADL). Additional secondary outcomes included the amyloid PET and the cerebrospinal fluid (CSF) biomarkers p181-tau, Aβ_42_ and Aβ_40_. Data for blood biomarkers were not extracted due to the large heterogeneity in the type of biomarkers studied/reported across trials. Safety outcomes comprised amyloid-related imaging abnormalities (ARIA) with edema or effusions (ARIA-E), ARIA with cerebral microhemorrhages or superficial siderosis (ARIA-H), and all-cause mortality.

### Risk of bias and certainty in evidence

Quality assessment of the included studies was performed for CDR-SB using the revised Cochrane Collaboration’s Risk of Bias Tool (RoB 2.0)^12^. Overall risk of bias was considered low if all individual domains indicated low risk of bias, high if at least one domain indicated high risk of bias, and of some concerns in any other case. Moreover, we used the Grading of Recommendations Assessment, Development and Evaluation (GRADE) framework to rate the certainty of evidence in effect estimates for CDR-SB^13^. Finally, we explored the presence of small-study effects for CDR-SB visually by a funnel plot and formally with the Egger’s test^14^.

### Data synthesis and analysis

For continuous data, we calculated Hedges’ g (a standard measure of standardized mean differences (SMDs)) with 95% confidence intervals (CIs). The use of this standardized metric allowed for pooling of effect sizes derived from measures/scales that may be slightly different across studies. Examples of this include pooling of different versions of ADAS-Cog (e.g.,^11–14^); also, pooling of amyloid PET scan results using different tracers (PiB, florbetaben, florbetapir, or flutemetamol) and quantification techniques (SUVr and centiloids). Meta-analyses were conducted applying a random-effects model^15^. To calculate the between-study heterogeneity (tau-squared, *t*^2^), we used the Restricted Maximum Likelihood (REML) estimator^16^. To calculate the confidence interval around the pooled effect, we used the Knapp-Hartung adjustments^17^. For dichotomous outcomes, we calculated risk ratios (RRs) with 95% CIs and performed meta-analyses using the Mantel–Haenszel method^18^. For all meta-analyses, statistical heterogeneity across studies was assessed with the *I*^2^ statistic, with values more than 50% indicating substantial heterogeneity^19^. For statistically significant results of continuous outcomes, we calculated numbers needed to treat (NNT) using the Kraemer & Kupfer’s method which calculates NNT directly from the SMD using the area under the curve (AUC)^20^.

Given the possibility for variable effects of different doses of individual mAbs (different doses of one mAb may have different binding affinities for Aβ forms and therefore mechanism of action^21^), we did not merge the different doses within a single trial, but handled them as separate units of analysis. To determine the overall effect of mAbs as a class, we conducted meta-analyses by combining all different drugs and doses for any given outcome. Then, to examine effects of individual mAbs, we performed subgroup analyses by individual mAbs. Additionally, since Aβ monomers could play a neuroprotective role in AD, we conducted subgroup analyses based on the relative binding affinity of mAbs for Aβ monomers^21^. To assess the clinical relevance of possible Aβ reductions induced by mAbs, we computed the Pearson’s r correlation coefficients between SMDs for change in amyloid PET and SMDs for the clinical outcomes CDR-SOB, ADAS-Cog, and ADCS-ADL. Similarly, to assess the clinical relevance of CSF p181-tau reductions, we computed Pearson’s r between SMDs for CSF p181-tau and SMDs for CDR-SOB, ADAS-Cog, and ADCS-ADL. Finally, to examine the pooled effects of mAbs that have shown some clinical benefit, we performed a sensitivity analysis including only the FDA-approved Aducanumab, Lecanemab, and Donanemab.

Meta-analyses were performed using R version 3.6.3 (R Core Team, Vienna, Austria) and the statistical packages “esc”, “dmetar”, “meta”, “metafor” and RevMan 5.4 (Nordic Cochrane Centre, Copenhagen, Denmark). This manuscript was cleared for publication according to NIH regulations.

## Results

**Fig. 1:**
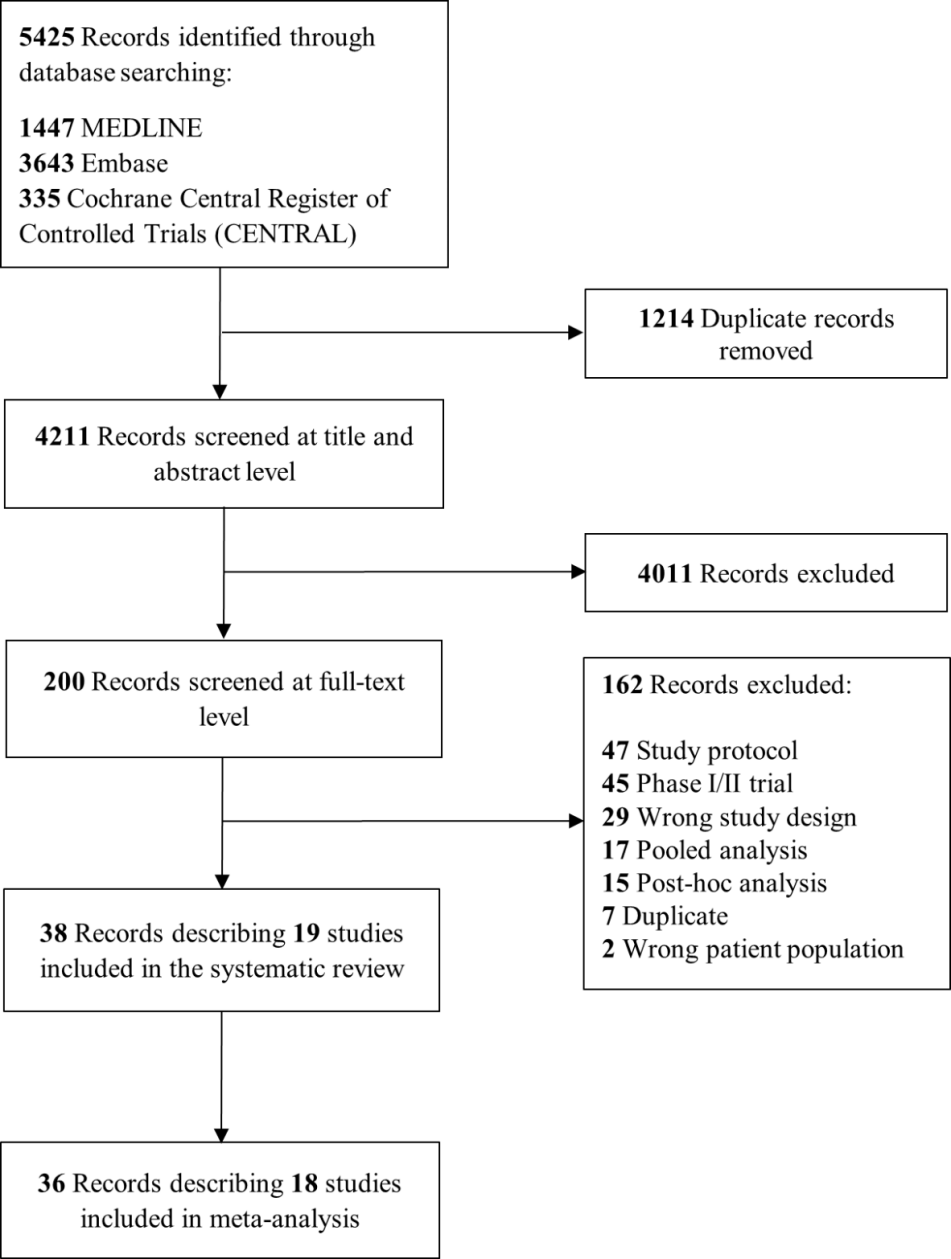
Flow diagram of study selection.

Our literature search identified 5425 records. Following deduplication and screening, 38 records describing 19 studies were included in the systematic review. Of those, 18 studies enrolling a total of 21,122 participants were designed as double-blinded, parallel-group, placebo-controlled trials and were included in the quantitative portion of our study, the meta-analysis (Fig. 1)^4–6,22–30^. Of the 19 studies included in the systematic review, four trials assessed Bapineuzumab^25,26^, four assessed Solanezumab^22–24^, three assessed Gantenerumab^28–30^, two assessed Crenezumab^27^, two assessed Aducanumab^5^, one assessed Donanemab^6^, and one assessed Lecanemab^4^. The one trial that was included in the systematic review but not in the meta-analysis compared Donanemab to Aducanumab and there was no placebo group^31^. One trial did not assess any clinical outcomes and was only included in the meta-analysis for the syntheses of biomarkers^29^. Of note, eight of the trials included in the present systematic review and meta-analysis were prematurely terminated due to either futility based on interim analysis or lack of clinical efficacy observed in other studies assessing the same mAb^5,26–29^. Study and patient baseline characteristics are shown in Table 1.

**Table 1:** Study and patients baselin﻿e characteristics.

Study ID	Duration of treatment (weeks)	Reason for study discontinuation	AD stage (MMSE score for inclusion)	Study arms included in the meta-analysis	No of patients randomized, n	Age (mean ± SD), years	Male, n (%)	Female, n (%)	*APOE* ε4 carrier, n (%)	CDR-SOB (mean ± SD)	ADAS-Cog^†^ (mean ± SD)	MMSE (mean ± SD)
Doody 2014 (EXPEDITION 1, NCT00905372)	80	N/A	Mild-to-moderate (16–26)	Solanezumab 400 mg IV Q4W	506	75.0 ± 7.9	207 (40.9)	299 (59.1)	266 (57.3) (tested in a subset of 464 patients)	NR	22.0 ± 8.0	21.0 ± 4.0
				Placebo	506	74.4 ± 8.0	219 (43.3)	287 (56.7)	288 (61.3) (tested in a subset of 470 patients)	NR	22.0 ± 9.0	21.0 ± 3.0
Doody 2014 (EXPEDITION 2, NCT00812565)	80	N/A	Mild-to-moderate (16–26)	Solanezumab 400 mg IV Q4W	521	72.5 ± 8.0	238 (45.7)	283 (54.3)	263 (56.8) (tested in a subset of 463 patients)	NR	24.0 ± 9.0	21.0 ± 3.0
				Placebo	519	72.4 ± 7.8	233 (44.9)	286 (55.1)	281 (59.5) (tested in a subset of 472 patients)	NR	23.0 ± 10.0	21.0 ± 3.0
Honig 2018 (EXPEDITION 3, NCT01900665)	80	N/A	Mild (20–26)	Solanezumab 400 mg IV Q4W	1057	72.7 ± 7.8	457 (43.2)	600 (56.8)	712 (69.3) (tested in a subset of 1027 patients)	3.9 ± 1.9	28.9 ± 8.3	22.8 ± 2.8
				Placebo	1072	73.3 ± 8.0	441 (41.1)	631 (58.9)	685 (66.3) (tested in a subset of 1033 patients)	3.9 ± 2.0	29.7 ± 8.5	22.6 ± 2.9
Sperling 2023 (A4, NCT02008357)	240	N/A	Prodromal AD (MMSE ≥ 25)	Solanezumab 1600 mg IV Q4W	564	72.0 ± 4.7	235 (41.7)	329 (58.3)	333 (59.0)	0.1 ± 0.2		28.8 ± 1.3
				Placebo	583	71.9 ± 5.0	231 (39.6)	352 (60.4)	342 (58.7)	0.0 ± 0.2		28.8 ± 1.2
Salloway 2014 (Bapineuzumab 301, NCT00574132)	78	N/A	Mild-to-moderate (16–26)	Bapineuzumab 0.5 mg/kg IV Q13W	337	73.0 ± 9.5	162 (48.1)	175 (51.9)	0 (0.0)	NR	22.3 ± 9.6	21.2 ± 3.4
				Bapineuzumab 1.0 mg/kg IV Q13W	329	73.2 ± 9.4	143 (43.5)	186 (56.5)	0 (0.0)	NR	22.3 ± 9.9	21.2 ± 3.3
				placebo	524	71.8 ± 10.1	258 (49.2)	266 (50.8)	0 (0.0)	NR	22.1 ± 10.1	21.3 ± 3.2
Salloway 2014 (Bapineuzumab 302, NCT00575055)	78	N/A	Mild-to-moderate (16–26)	Bapineuzumab 0.5 mg/kg IV Q13W	673	72.1 ± 8.1	304 (45.2)	369 (54.8)	673 (100.0)	NR	23.6 ± 9.5	20.7 ± 3.1
				placebo	448	72.4 ± 8.3	196 (43.7)	252 (56.3)	448 (100.0)	NR	23.9 ± 9.8	20.7 ± 3.2
Vandenberghe 2016 (Bapineuzumab 3000, NCT00667810)	78	Lack of clinical efficacy observed in Bapineuzumab 301 and 302 studies	Mild-to-moderate (16–26)	Bapineuzumab 0.5 mg/kg IV Q13W	267	71.4 ± 9.4	116 (43.4)	151 (56.6)	0 (0.0)	NR	23.2 (10.0)	20.8 ± 3.2
				Bapineuzumab 1.0 mg/kg IV Q13W	263	70.8 ± 9.7	113 (43.0)	150 (57.0)	0 (0.0)	NR	23.5 (9.3)	20.8 ± 3.1
				Placebo	344	69.9 ± 9.8	145 (42.2)	199 (57.8)	0 (0.0)	NR	22.9 (10.2)	20.8 ± 3.1
Vandenberghe 2016 (Bapineuzumab 3001, NCT00676143)	78	Lack of clinical efficacy observed in Bapineuzumab 301 and 302 studies	Mild-to-moderate (16–26)	Bapineuzumab 0.5 mg/kg IV Q13W	654	71.0 ± 7.7	233 (35.6)	421 (64.4)	654 (100.0)	NR	23.2 (8.9)	20.9 ± 3.1
				Placebo	439	70.3 ± 7.8	177 (40.3)	262 (59.7)	439 (100.0)	NR	22.6 (8.9)	21.0 ± 3.0
Ostrowitzki 2022 (CREAD, NCT02670083)	105	Futility based on interim analysis	Prodromal-to-mild (≥ 22)	Crenezumab 60 mg/kg IV Q4W	404	71.0 ± 7.9	168 (41.6)	236 (58.4)	293 (72.7)	3.9 ± 1.7	29.4 ± 7.6	23.7 ± 3.0
				Placebo	409	70.3 ± 8.4	162 (39.6)	247 (60.4)	292 (71.7)	3.8 ± 1.6	28.9 ± 7.4	23.4 ± 2.9
Ostrowitzki 2022 (CREAD2, NCT03114657)	105	Futility based on interim analysis	Prodromal-to-mild (≥ 22)	Crenezumab 60 mg/kg IV Q4W	407	71.1 ± 7.5	176 (43.2)	231 (56.8)	271 (66.9)	3.7 ± 1.6	28.8 ± 7.4	23.6 ± 2.8
				Placebo	399	70.7 ± 7.9	174 (43.6)	225 (56.4)	263 (65.9)	3.8 ± 1.6	28.9 ± 7.3	23.5 ± 2.9
Ostrowitzki 2017 (Scarlet RoAD, NCT01224106)	104	Futility based on interim analysis	Prodromal (≥ 24)	Gantenerumab 105 mg SC Q4W	271	70.3 ± 7.0	119 (43.9)	152 (56.1)	151 (79.0) (tested in a subset of 191 patients)	2.2 ± 1.0	23.1 ± 6.9	25.7 ± 2.3
				Gantenerumab 225 mg SC Q4W	260	71.3 ± 7.1	108 (41.5)	152 (58.5)	112 (61.5) (tested in a subset of 182 patients)	2.0 ± 0.9	23.0 ± 6.2	25.7 ± 2.2
				Placebo	266	79.5 ± 7.5	117 (44.0)	149 (56.0)	131 (70.3) (tested in a subset of 186 patients)	2.1 ± 1.0	23.5 ± 7.2	25.7 ± 2.1
Voyle 2018 (Marguerite RoAD, NCT02051608)	104	Futility based on interim analysis	Mild (20–26)	Gantenerumab 105–225 mg SC Q4W	192	69.7 ± 8.9	94 (49.0)	98 (51.0)	NR	NR	NR	NR
				Placebo	195	70.1 ± 8.6	82 (42.1)	113 (57.9)	NR	NR	NR	NR
Bateman 2023 (GRADUATE I, NCT03444870)	116	N/A	Prodromal-to-mild (≥ 22)	Gantenerumab 510 mg SC Q2W	499	61.1 ± 7.9	209 (41.9)	290 (58.1)	326 (65.3)	3.7 ± 1.7	28.1 ± 7.1	23.5 ± 3.3
				placebo	485	72.1 ± 7.8	230 (47.4)	255 (52.6)	328 (67.6)	3.7 ± 1.6	28.1 ± 6.8	23.6 ± 3.0
Bateman 2023 (GRADUATE II, NCT03443973)	116	N/A	Prodromal-to-mild (≥ 22)	Gantenerumab 510 mg SC Q2W	498	71.6 ± 7.8	210 (42.2)	288 (57.8)	333 (66.9)	3.7 ± 1.6	28.1 ± 6.9	23.6 ± 3.1
				Placebo	477	71.8 ± 7.4	192 (40.3)	285 (59.7)	321 (67.2)	3.5 ± 1.5	28.2 ± 7.0	23.8 ± 3.2
Haeberlein 2022 (EMERGE, NCT02484547)	78	Futility based on interim analysis	Prodromal-to-mild (24–30)	Aducanumab 3 mg/kg (*APOE* ε4^+^) or 6 mg/kg (*APOE* ε4^-^) IV Q4W	543	70.6 ± 7.4	274 (50.5)	269 (49.5)	362 (67.0)	2.5 ± 1.0	22.5 ± 6.8	26.3 ± 1.7
				Aducanumab 6 mg/kg (*APOE* ε4^+^) or 10 mg/kg (*APOE* ε4^-^) IV Q4W	547	70.6 ± 7.5	263 (48.1)	284 (51.9)	365 (67.0)	2.5 ± 1.1	22.3 ± 7.1	26.3 ± 1.7
				Placebo	548	70.8 ± 7.4	258 (47.1)	290 (52.9)	368 (67.0)	2.5 ± 1.0	21.9 ± 6.7	26.4 ± 1.8
Haeberlein 2022 (ENGAGE, NCT02477800)	78	Futility based on interim analysis	Prodromal-to-mild (24–30)	Aducanumab 3 mg/kg (*APOE* ε4^+^) or 6 mg/kg (*APOE* ε4^-^) IV Q4W	547	70.4 ± 7.0	263 (48.1)	284 (51.9)	391 (71.0)	2.4 ± 1.0	22.5 ± 6.3	26.4 ± 1.8
				Aducanumab 6 mg/kg (*APOE* ε4^+^) or 10 mg/kg (*APOE* ε4^-^) IV Q4W	555	70.0 ± 7.7	263 (47.4)	292 (52.6)	378 (68.0)	2.4 ± 1.0	22.4 ± 6.5	26.4 ± 1.8
				Placebo	544	69.8 ± 7.7	258 (47.3)	286 (52.7)	376 (69.0)	2.4 ± 1.0	22.5 ± 6.6	26.4 ± 1.7
Sims 2023 (TRAILBLAZER-ALZ 2, NCT04437511)	76	N/A	Prodromal-to-mild AD (MMSE: 20–28)	Donanemab 1400 mg IV Q4W	860	73.0 ± 6.2	367 (42.7)	493 (57.3)	598 (69.8)	4.0 ± 2.1	28.7 ± 8.8	22.4 ± 3.8
				Placebo	876	73.0 ± 6.2	373 (42.6)	503 (57.4)	621 (71.2)	3.9 ± 2.1	29.3 ± 8.9	22.2 ± 3.9
Salloway 2022 (TRAILBLAZER-ALZ 4, NCT05108922)^§^	76	N/A	20–30	Donanemab 1400 mg IV Q4W	71	74.1 ± 6.9	33 (46.5)	38 (53.5)	49 (69.0)	NR	NR	25.0 ± 2.7
				Aducanumab 10 mg/kg IV Q4W	69	72.7 ± 6.8	27 (39.1)	42 (60.9)	49 (71.0)	NR	NR	24.4 ± 3.0
van Dyck 2022 (CLARITY AD, NCT03887455)	72 weeks	N/A	Prodromal-to-mild (24–30)	Lecanemab 10 mg/kg IV Q2W	859	71.4 ± 7.9	416 (48.4)	443 (51.6)	592 (68.9)	3.2 ± 1.3	24.5 ± 7.1	25.5 ± 2.2
				placebo	875	71.0 ± 7.8	411 (47.0)	464 (53.0)	600 (68.6)	3.2 ± 1.3	24.4 ± 7.6	25.6 ± 2.2

The detailed risk of bias assessment is summarized in Table S3. Overall, nine studies were at low risk of bias^4,6,22,24,25,30^, one at high risk^27^, and seven were deemed as having some concerns^5,23,26–28^. Missing outcome data was the most common domain for assigning some degree of risk of bias. One trial did not assess any clinical outcomes and was not included in the risk of bias assessment^29^.

### Primary outcome

**Fig. 2:**
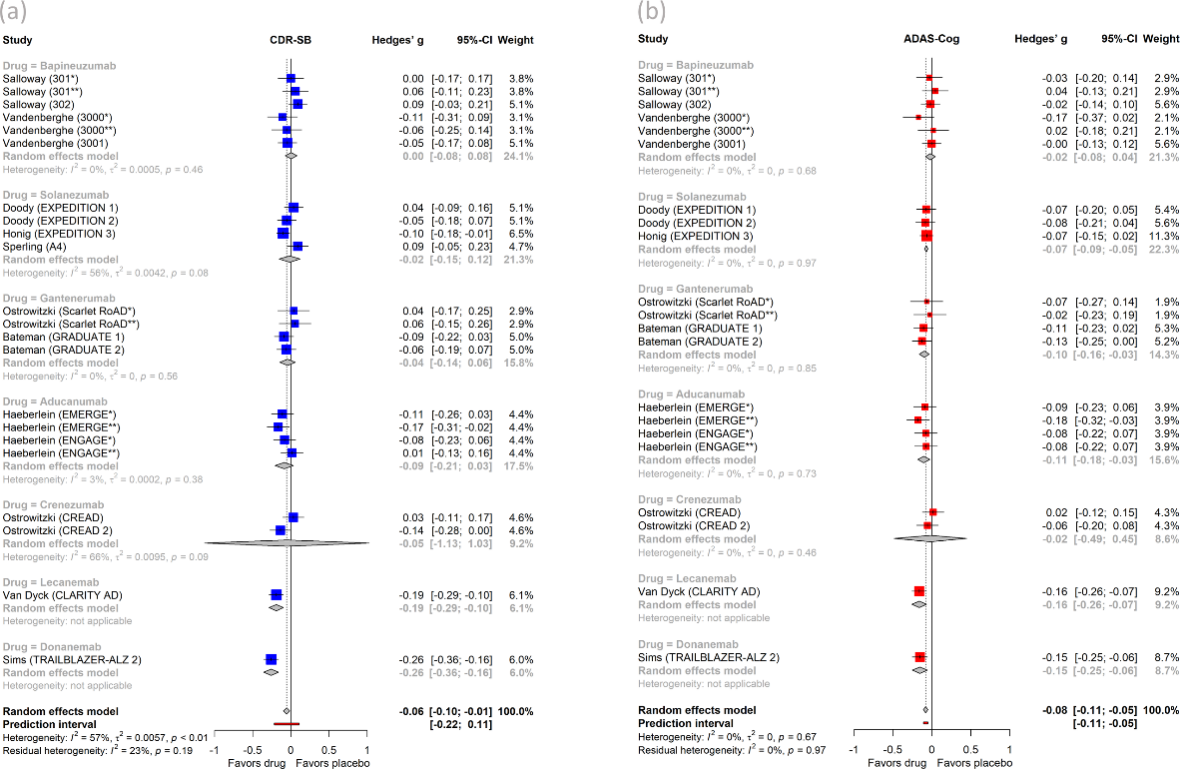
Forest plots of (**a**) Clinical Dementia Rating Scale-Sum of Boxes (CDR-SB) and (**b**) Alzheimer’s Disease Assessment Scale-Cognitive Subscale (ADAS-Cog) meta-analyses with subgroup analyses by individual drug.

Compared to placebo, mAbs attenuated worsening on CDR-SB [SMD =  − 0.06 (95% CI, − 0.10 to − 0.01, *I*^2^ = 57%)] (Fig. 2a). Although statistically significant, the effect size for this beneficial difference was very small (< 0.2) and corresponded to a NNT of 30. The pooled effect size corresponded to a point-change on CDR-SB was much smaller than the clinically meaningful change as currently defined in the literature (Table 2)^32^.

**Table 2:** Clinical outcome effect size interpretation, Numbers Needed to Treat, and clinical meaningfulness.

Clinical measure	Drug(s) included in meta-analysis or subgroup analysis that reached statistical significance	Effect size (Standardized mean difference)	Size of the effect	Number Needed to Treat (NNT)	Point-change approximation on original scale	Minimal score change considered clinically important^31^
CDR-SB	Bapineuzumab, Solanezumab, Gantenerumab, Aducanumab, Crenezumab, Lecanemab, Donanemab	−0.06	Very small	30	< 0.70	1
	Lecanemab	−0.19	Small	9	< 0.70	1
	Donanemab	−0.26	Small to moderate	7	0.70	1
ADAS-Cog	Bapineuzumab, Solanezumab, Gantenerumab, Aducanumab, Crenezumab, Lecanemab, Donanemab	−0.08	Very small	22	< 1.44	2
	Solanezumab	−0.07	Very small	25	< 1.44	2
	Gantenerumab	−0.10	Very small	18	< 1.44	2
	Aducanumab	−0.11	Very small	16	< 1.44	2
	Lecanemab	−0.16	Small	11	1.44	2
	Donanemab	−0.15	Small	12	< 1.44	2
ADCS-ADL	Solanezumab, Gantenerumab, Aducanumab, Crenezumab, Lecanemab, Donanemab	0.09	Very small	20	< 2	-
	Aducanumab	0.13	Very small	14	< 2	-
	Lecanemab	0.25	Small to moderate	7	2	-
	Donanemab	0.18	Small	10	< 2	-
MMSE	Bapineuzumab, Solanezumab, Gantenerumab, Aducanumab, Crenezumab, Donanemab	0.05	Very small	35	< 0.8	2
	Solanezumab	0.08	Very small	22	0.8	2

The Egger’s test in the meta-analysis of the primary outcome was statistically significant (*p* = 0.03). However, we have no concerns for publication bias because inspection of the funnel plot (Figure S1) shows that asymmetry is driven by Lecanemab and Donanemab effect sizes on the left side of the funnel. Lecanemab and Donanemab are known outliers in our analysis producing by far the most robust clinical effects (see subgroup analyses by drug) and are expected to cause asymmetry in the funnel. Furthermore, it is known that funnel plot asymmetry can sometimes occur by chance. The quality of evidence by GRADE was moderate and mainly downgraded for risk of bias (Table S4).

### Secondary outcomes

Compared to placebo, mAbs attenuated worsening on ADAS-Cog [SMD =  − 0.08 (95% CI, − 0.11 to − 0.05, *I*^2^ = 0%)] (Fig. 2b), ADCS-ADL [SMD = 0.09 (95% CI, 0.03 to 0.14, *I*^2^ = 61%)] (Figure S2b), and MMSE [SMD = 0.05 (95% CI, 0.02 to 0.08, *I*^2^ = 0%)] (Figure S2a). The effect sizes for these improvements were very small (< 0.2) and corresponded to NNTs of 22, 20, and 35, respectively. The pooled effect sizes for ADAS-Cog, ADCS-ADL, MMSE corresponded to point-changes that are below the thresholds for clinically meaningful change (Table 2)^32^.

**Fig. 3:**
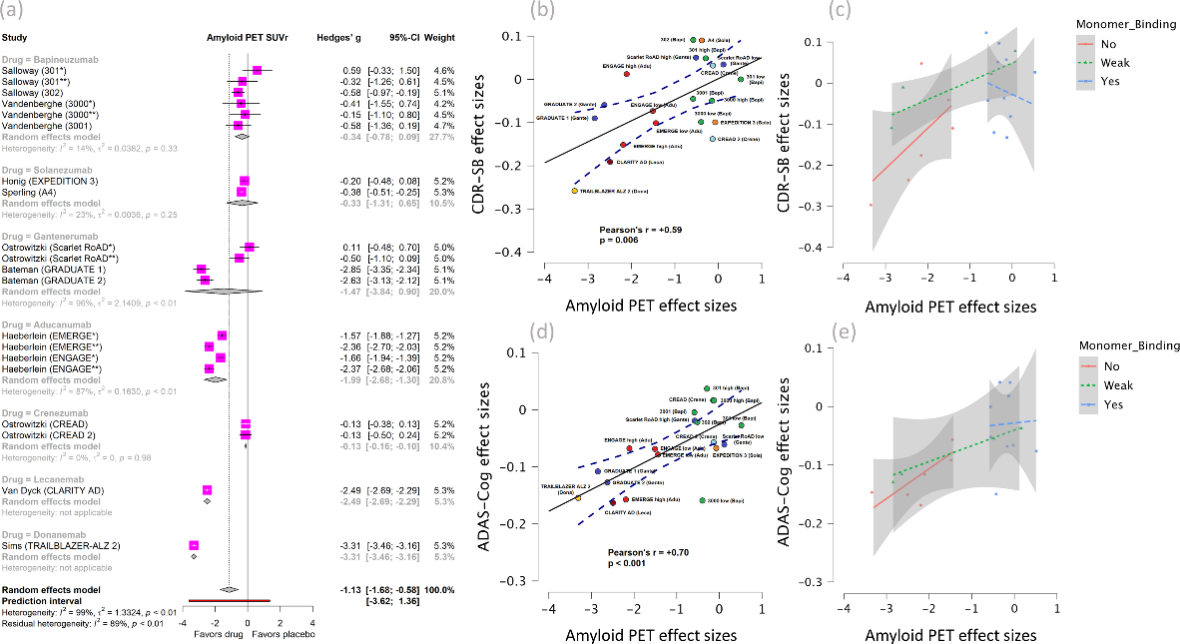
(**a**) Forest plot of amyloid PET with subgroup analysis by individual drug. (**b**) Correlation graph between effect sizes of amyloid PET and CDR-SB. (**c**) Correlation between amyloid PET and CDR-SB with subgroups based on binding affinity for Aβ monomers. (**d**) Correlation graph between effect sizes of amyloid PET and ADAS-Cog. (**e**) Correlation between amyloid PET and ADAS-Cog with subgroups based on binding affinity for Aβ monomers.

Compared to placebo, mAbs significantly reduced amyloid on PET [SMD =  − 1.13 (95% CI, − 1.68 to − 0.58 *I*^2^ = 99%) (Fig. 3a). The effect size for this reduction was very large (> 0.8). Monoclonal antibodies also reduced CSF p181-tau [SMD =  − 0.44 (95% CI, − 0.60. to − 0.28, *I*^2^ = 51%) (Figure S3), with the effect size being moderate (~ 0.5). Antibodies increased CSF Aβ_42_ [SMD = 1.12 (95% CI, 0.64 to 1.61, *I*^2^ = 91%) (Figure S4) and Aβ_40_ [SMD = 0.7 (95% CI, 0.15 to 1.26, *I*^2^ = 90%) (Figure S5) by a very large and moderate-to-large effect size, respectively.

**Fig. 4:**
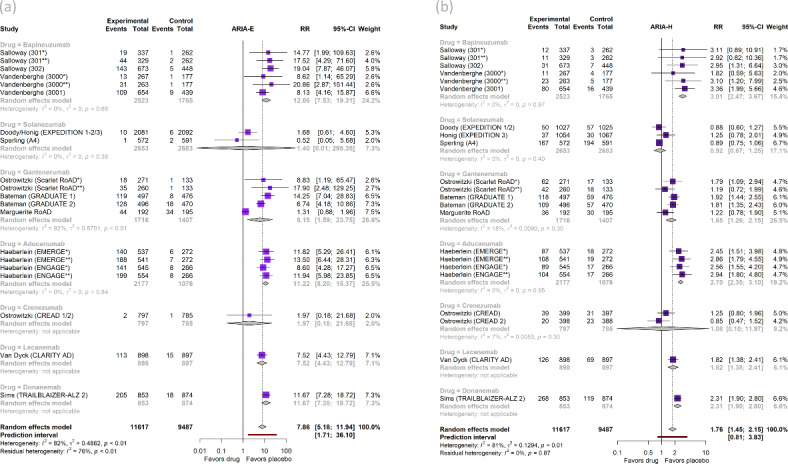
Forest plots of (**a**) Amyloid-Related Imaging Abnormalities with edema/effusion (ARIA-E) and (**b**) ARIA with microhemorrhages or superficial siderosis (ARIA-H) with subgroup analyses by individual drug.

Regarding ARIA, compared to placebo, mAbs significantly increased risk of ARIA-E [RR = 7.86 (95% CI, 5.18 to 11.94), *I*^2^ = 82%)] (Fig. 4a) by a very large effect size and ARIA-H [RR = 1.76 (95% CI, 1.45 to 2.15, *I*^2^ = 81%)] (Fig. 4b) by a moderate effect size. Finally, all-cause mortality did not differ between mAbs and placebo [RR = 0.91 (95% CI, 0.68 to 1.23, *I*^2^ = 18%)] (Figure S6).

### Subgroup analyses by drug

Subgroup analyses by individual drugs are shown in Figs. 2–4 and Supplementary material (S2-S6). Among individual drugs, Donanemab induced the largest benefit on the primary clinical outcome CDR-SB [SMD =  − 0.26 (95% CI, − 0.36 to − 0.16)] which was in the effect size range of small to moderate (0.2–0.5) (Fig. 2a). Despite statistical significance, this benefit corresponded to 0.70 points on CDR-SB scale which is smaller than the threshold of 1 point which has been considered a clinically meaningful change on this measure (Table 2). For other clinical outcomes, Donanemab improved MMSE by a very small effect size (0.11), ADAS-Cog by a very small to small effect size (0.15), and ADCS-ADL by a small effect size (0.18) (Figs. 2b, S2). All benefits by Donanemab corresponded to point-differences between drug and placebo that are below the threshold for clinically important changes (Table 2).

Lecanemab attenuated worsening on the primary clinical outcome CDR-SB by a small effect size [SMD =  − 0.19 (95% CI, − 0.29 to − 0.10)] (Fig. 2a). Lecanemab also attenuated worsening on other clinical outcomes including ADAS-Cog by a small effect size (~ 0.2) and ADCS-ADL by a small to moderate effect size (0.25) (Figs. 2b, S2b). All benefits by Lecanemab corresponded to point-differences between drug and placebo which were below the threshold of clinically important changes (Table 2).

Aducanumab attenuated worsening on the clinical outcomes ADAS-Cog and ADCS-ADL only by very small effect sizes (< 0.2) (Figs. 2b, S2b). Solanezumab attenuated worsening on ADAS-Cog and MMSE by very small effect sizes (< 0.2) (Figs. 2b, S2a). Similarly, Gantenerumab attenuated worsening on ADAS-Cog by very a small effect size (< 0.2) (Fig. 2b). These benefits on clinical measures by individual drugs were also below the threshold of clinically important changes (Table 2).

For biomarker outcomes, Aducanumab, Lecanemab, and Donanemab induced very large reductions on amyloid PET (Fig. 2a) by effect sizes (> 0.8). Aducanumab and Lecanemab reduced CSF p181-tau by a large effect size (~ 0.8). Gantenerumab decreased CSF p181-tau by moderate to large effect size (~ 0.6), while Bapineuzumab by a small to moderate effect size (0.3) (Figure S3). Moreover, for other CSF amyloid biomarkers, Lecanemab, Aducanumab, and Crenezumab increased Aβ_42_ by very large effect sizes (Figure S4), whereas only the latter increased Aβ_40_, also by a very large effect size (Figure S5).

Regarding ARIA, Aducanumab, Lecanemab, Donanemab, Gantenerumab, and Bapineuzumab increased the incidence of ARIA-E by a very large effect size (RR > 2) (Fig. 4a). Bapineuzumab, Aducanumab, and Donanemab increased ARIA-H by a very large effect size (RR > 2) and Lecanemab and Gantenerumab by moderate effect size (RR 1.2–2) (Fig. 4b). Solanezumab and Crenezumab did not increase any of the ARIA-E or ARIA-H risks compared to placebo. Finally, the risk of all-cause mortality did not increase with any drug compared to placebo (Figure S6).

### Subgroup analyses by binding affinity for Aβ monomers

In terms of their binding affinity for Aβ monomers, mAbs fall into one of the following three subgroups: no binding, weak binding, and strong binding. The subgroup of mAbs not binding to monomers included drugs that preferentially bind to proto-fibrils (Lecanemab), fibrils (Aducanumab) or the Aβ plaque itself (Donanemab)^4,21^. The subgroup of mAbs exhibiting weak binding to monomers included Gantenerumab which in addition to some binding to monomers, it has affinity for oligomers and fibrils^4,21^. The subgroup with strong binding to monomers included mAbs that bind either only to monomers (Solanezumab) or to monomers but also to all other Aβ forms (Bapineuzumab, Crenezumab)^21^.

Forest plots of subgroup analyses by binding affinity are shown in Figures S7-S13. Antibodies not binding to monomers attenuated worsening on CDR-SB compared to placebo, however by a very small effect size [SMD =  − 0.14 (95% CI, − 0.24 to − 0.04, *I*^2^ = 56%)]. On the contrary, antibodies with weak or strong binding affinity for monomers did not produce any significant effects on CDR-SB. (Figure S7a).

Antibodies not binding to monomers attenuated worsening on ADAS-Cog compared to placebo by a very small effect size [SMD =  − 0.13 (95% CI, − 0.18 to − 0.09, *I*^2^ = 0%)], followed by a smaller beneficial effect of mAbs binding weakly to monomers [SMD =  − 0.10 (95% CI, − 0.17 to − 0.03, *I*^2^ = 0%)], followed by an even smaller (marginal) beneficial effect of mAbs binding strongly to monomers [SMD =  − 0.04 (95% CI, − 0.08 to − 0.01, *I*^2^ = 0%)] (Figure S7b).

For ADCS-ADL, there was a favorable difference from placebo for the mAb subgroup not binding to monomers [SMD = 0.17 (95% CI, 0.10 to 0.24, *I*^2^ = 17%)] of marginally small (~ 0.2) effect size, but not for mAbs binding weakly or strongly to monomers (Figure S8b). None of the three subgroups induced a statistically significant effect on MMSE score (Figure S8a).

Regarding biomarker outcomes, mAbs not binding to monomers produced a significant amyloid reduction on PET compared to placebo [SMD =  − 2.30 (95% CI, − 2.97 to − 1.63, *I*^2^ = 97%)] of very large (> 0.8) effect size, followed by a substantially smaller reduction induced by mAbs binding strongly to monomers [SMD =  − 0.28 (95% CI, − 0.43 to − 0.13, *I*^2^ = 16%)] which was of small to moderate (0.2–0.5) effect size (Figure S9a).

In addition, mAbs not binding to monomers produced a large-effect size reduction of CSF p181-tau compared to placebo [SMD =  − 0.81 (95% CI, − 1.08 to − 0.55, *I*^2^ = 0%)], followed by a smaller reduction by mAbs binding weakly to monomers [SMD =  − 0.57 (95% CI, − 0.85 to − 0.29, *I*^2^ = 0%)] which was of moderate to large effect size, followed by an even smaller reduction by mAbs binding strongly to monomers [SMD =  − 0.26 (95% CI, − 0.41 to − 0.10, *I*^2^ = 0%)] which was of small to moderate effect size (Figure S9b).

Antibodies not binding to monomers produced a very large effect size increase of CSF Aβ_42_ compared to placebo [SMD = 1.27 (95% CI, 0.77 to 1.77, *I*^2^ = 61%)], similarly to mAbs binding strongly to monomers [SMD = 1.43 (95% CI, 0.58 to 2.29, *I*^2^ = 93%)], with mAbs binding weakly to monomers having no effect [SMD = 0.33 (95% CI, − 0.36 to 1.02, *I*^2^ = 83%)] (Figure S10). Finally, antibodies binding strongly to monomers was the only subgroup that changed (increased) CSF Aβ_40_ [SMD = 1.01 (95% CI, 0.46 to 1.56, *I*^2^ = 84%)] and it did so by a very large effect size (Figure S11).

The highest incidence of ARIA-E was observed for mAbs not binding to monomers [RR = 9.61 (95% CI, 5.52 to 16.72, *I*^2^ = 30%)], followed by mAbs binding strongly [RR = 8.37 (95% CI, 3.66 to 19.16, *I*^2^ = 58%)] and then weakly to monomers [RR = 6.15 (95% CI, 1.59 to 23.75, *I*^2^ = 92%)] (Figure S12a). For ARIA-H, the highest risk compared to placebo was observed for mAbs not binding to monomers [RR = 2.29 (95% CI, 1.94 to 2.71, *I*^2^ = 0%)], followed by mAbs binding weakly [RR = 1.65 (95% CI, 1.26 to 2.15, *I*^2^ = 18%)], followed by mAbs binding strongly to monomers [RR = 1.55 (95% CI, 1.05 to 2.28, *I*^2^ = 76%)] (Figure S12b). Mortality risk was similar between mAbs and placebo across all subgroups (Figure S13).

### Association of Aβ and p181-tau reductions with benefits on clinical measures

The amyloid reduction on PET was moderately correlated with the reductions of CDR-SB (correlation between SMDs, r =  + 0.59, *p* = 0.006) and ADAS-Cog (r =  + 0.70, *p* < 0.001) (Fig. 3b, 3d). Notably, these correlations were mostly driven by mAbs with no binding affinity for Aβ monomers (Fig. 3c, e).

Similarly, the reduction of CSF p181-tau was moderately correlated with the reduction of ADAS-Cog (r =  + 0.59, *p* = 0.022) and strongly correlated with the reduction of ADCS-ADL (r =  − 0.79, *p* = 0.035). These correlations were mostly driven by mAbs with no binding affinity to Aβ monomers.

### Sensitivity analysis of FDA-approved mAbs

The FDA-approved mAbs (Aducanumab, Lecanemab, Donanemab) combined showed attenuation of worsening on CDR-SB [SMD =  − 0.14 (95% CI, − 0.24 to − 0.04, *I*^2^ = 56%)] (Figure S14a) and ADAS-Cog [SMD =  − 0.13 (95% CI, − 0.18 to − 0.09, *I*^2^ = 0%)] (Figure S14b). Although these benefits were greater than those induced by all mAbs combined (0.14 vs 0.06 and 0.13 vs 0.08, respectively), the effect sizes were still very small (< 0.2). Moreover, FDA-approved mAbs showed a benefit on ADCS-ADL [SMD = 0.17 (95% CI, 0.10 to 0.24, *I*^2^ = 17%)] (Figure S14c) with an effect size in the range of small (0.17) which was larger compared to the very small effect (0.09) induced by all mAbs combined. MMSE was only assessed in Aducanumab and Donanemab studies with their pooled effect being statistically non-significant [SMD = 0.06 (95% CI, − 0.03 to 0.14, *I*^2^ = 6%)] (Figure S14d); on the contrary, the pooled effect of all mAbs combined revealed a statistically significant benefit on MMSE, albeit of very small effect size (0.05) (Figure S2a).

For amyloid PET, the three FDA-approved mAbs induced robust amyloid reduction [SMD =  − 2.30 (95% CI, − 2.97 to − 1.63 *I*^2^ = 97%)] (Figure S15a), with an effect size larger than the one produced by all mAbs combined (2.30 vs 1.13). Moreover, only Aducanumab and Lecanemab studies assessed CSF p181-tau and Aβ_42_, inducing a combined decrease of SMD =  − 0.81 (95% CI, − 1.08 to − 0.55, *I*^2^ = 0%) (Figure S15b) and an increase of SMD = 1.27 (95% CI, 0.77 to 1.77, *I*^2^ = 61%) (Figure S15c) respectively; the effect sizes for these changes were larger compared to those induced by all mAbs combined (0.81 vs 0.44 and 1.27 vs 1.12,respectively).

Of note, FDA-approved mAbs substantially increased the risk of ARIA-E [RR = 10.36 (95% CI, 8.12 to 13.21), *I*^2^ = 0%)] (Figure S16a) and ARIA-H [RR = 2.29 (95% CI, 1.94 to 2.71), *I*^2^ = 0%)] (Fig. 16b), with the RRs being larger than those found in analyses of all mAbs combined (10.36 vs 7.86 and 2.29 vs 1.76, respectively). Mortality risk for the three antibodies together did not differ from placebo (Figure S17).

## Discussion

In this systematic review and meta-analysis, we synthesized all available data from phase III RCTs on efficacy, target engagement, and safety of anti-Aβ mAbs in sporadic AD. Overall, we found that anti-Aβ mAbs as a class induced statistically significant but very small effect-size benefits on the clinical measures CDR-SB, ADAS-Cog, and ADCS-ADL. In addition, mAbs reduced amyloid burden measured by PET by a large effect size and this reduction was moderately correlated with beneficial differences for CDR-SB and ADAS-Cog. Moreover, there was a moderate effect size reduction of CSF p181-tau, which was moderately correlated with beneficial differences for ADAS-Cog and strongly correlated with beneficial differences for ADCS-ADL. However, mAbs also increased incidence of ARIA-E by a very large and ARIA-H by a moderate effect size.

Regarding individual mAbs, Donanemab and Lecanemab, followed by Aducanumab, produced the largest beneficial differences compared to placebo for clinical and biomarker outcomes. However, these statistically significant benefits were below the threshold for clinically meaningful change based on previous literature^32^.

Trying to explain why most clinical trials on mAbs against amyloid have historically failed to yield positive clinical results, many researchers hypothesized that it could be because trials included AD patients at a progressed stage of the disease in whom amyloid clearance may not be beneficial. For example, from 2014 to 2016, the first six phase III trials on mAbs (EXPEDITION 1 & 2 on Solanezumab; 301, 302, 3000, 3001 studies on Bapineuzumab) were conducted in patients with mild to moderate AD resulting in no significant differences between drug and placebo^24,25^. From that time to 2021–2022, a plethora of clinical trials on various mAbs (EXPEDITION 3 on Solanezumab; Scarlet RoAD on Gantenerumab; CREAD 1 and 2 on Crenezumab; EMERGE and ENGAGE on Aducanumab)^5,22,26,27^ included patients at earlier stages of the disease (prodromal and/or mild AD). Apart from Aducanumab which yielded statistically significant but clinically questionable results^5^, most studies were considered completely negative. Taking into consideration all these studies from 2014 to 2022, we previously showed via meta-regression that disease stage was not associated with the outcome produced by mAbs^33^. Since that time, more trials including prodromal and/or mild AD patients have been conducted (A4 on Solanezumab, CLARITY AD on Lecanemab, GRADUATE I and II on Gantenerumab, TRAILBLAZER 2 on Donanemab),^4,6,23^ showing that starting treatment with mAbs at an early disease stage did not result in better outcomes. For example, although Lecanemab and Donanemab trials were conducted in patients with prodromal to mild AD resulting in statistically significant benefits^4,6^, Solanezumab 2023 study was negative despite being conducted in prodromal AD only individuals^23^.

Accumulating evidence shows that the degree of amyloid plaque reduction is a better predictor of the clinical outcomes. In the present study, we showed that reduction in amyloid measured by PET was correlated with CDR-SB and ADAS-Cog improvements. Although it is plausible that amyloid reduction is one of the main factors that could determine mAbs efficacy, we hypothesized that there could be other factor(s) determining responses to mAbs. We hypothesized that the degree of binding to Aβ monomers plays a role in antibody responses, with greater monomeric binding leading to worse outcomes as monomers are suggested to play a protective physiological role in the brain^10^. We therefore performed subgroup analyses of all outcomes by the strength of binding affinity to monomers and showed the following: mAbs not binding to monomers (and preferentially binding to proto-fibrils or fibrils or plaques) induced statistically significant benefits consistently across multiple clinical and biomarker outcomes to a larger extent than mAbs binding weakly or strongly to monomers. Although results from subgroup analyses do not imply causation, our findings support the idea that preserving the monomer Aβ pool while targeting proto-fibrils, fibrils, and plaques might be beneficial in AD.

Overall, we updated and expanded upon the results of earlier meta-analyses^33,34^ and reached novel conclusions by conducting meta-analyses on eleven different outcomes, which collectively assessed effects on cognition, functional ability, amyloid and tau biomarkers, risk of ARIA and mortality. Furthermore, we showed there is a correlation between biomarker and clinical outcome improvements. Importantly, in addition to performing subgroup analysis by individual mAbs, we conducted a novel subgroup analysis based on binding affinity of mAbs for Aβ monomers. Results of this analysis may provide an explanation of the differences in effects of different mAbs and can inform the future development of anti-Aβ mAbs in AD.

Nevertheless, some limitations of our study should also be acknowledged. Meta-analyses of several biomarker outcomes were characterized by substantial statistical heterogeneity. In some cases, such as amyloid PET meta-analysis, heterogeneity was attributed to true mAb effect differences. More specifically, several mAbs effectively cleared brain amyloid whereas others did not, thereby contributing to highly heterogeneous effects which explains statistical heterogeneity. In contrast, heterogeneity of CSF Aβ_42_ and Aβ_40_ meta-analyses could have been partially due to true mAb differences, but it could also stem from the relative lack of validation and established universal cut-offs of analytical assays for amyloid measurement in CSF which can subsequently result in heterogeneous results even for the same drug at the same dose. Another limitation is that biomarker outcomes in the included trials were assessed in subpopulations of the study cohorts, therefore, our computed correlations between biomarker and clinical effects across studies level were performed under the assumption that the subpopulations were representative of the entire cohort in which the clinical outcomes were studied.

Our findings confirm that mAbs as a class, as well as some individual mAbs, are capable of robustly reducing amyloid burden; however, this effect is accompanied by statistically significant clinical improvements that are at the very best, of small-to-moderate effect size which questions the clinical meaningfulness of these effects. Recently, it was proposed that a 1-point change on CDR-SB, a 2-point change on ADAS-Cog (11 or 13), and a 2-point change on MMSE within a 12-month period could be considered the minimal changes for a statistical significant result to be clinically meaningful^32^. Aducanumab, Lecanemab, and Donanemab individually produced smaller point-changes on clinical measures than these benchmarks after being studied for 19.5 (Aducanumab) and 18 months (Lecanemab, Donanemab) in clinical trials, respectively. In our meta-analyses, we converted all statistically significant pooled effect sizes back to point changes on the original clinical scales and found that no individual drug or mAb subgroup reached the above thresholds for clinically meaningful change (Table 2). Therefore, based on these cut-offs, mAbs do not produce clinically important effects, therefore, their implementation in clinical practice remains questionable. However, the correlation between amyloid reduction and clinical improvements in our analysis supports the idea that amyloid is a rational target in AD. In addition, since mAbs demonstrably interfere with the underlying pathophysiology of AD, their benefit could be cumulative over time and their clinical effects larger in subsequent years^35^. Longer duration clinical trials and/or follow-ups may be needed to investigate this possibility. Our finding that mAbs leaving Aβ monomers intact while targeting proto-fibrils, fibrils, and plaques are associated with better clinical outcomes may direct focus of future drug development to mAbs with such targets.

## Conclusions

Our systematic review and meta-analysis demonstrate that mAbs are capable of robustly reducing brain amyloid burden and inducing statistically significant benefits on clinical measures that are of questionable clinical importance for the time period they were studied. The moderate correlation observed between amyloid reduction and beneficial effects on clinical measures suggests engagement of a pathophysiologically relevant target. Finally, we found evidence that mAbs with no affinity to monomers were more robustly associated with clinical and biomarker benefits.

## Electronic supplementary material

Below is the link to the electronic supplementary material.

Supplementary Material 1

## Data Availability

The datasets used and/or analysed during the current study are available from the corresponding author on reasonable request.

## References

[1] Karran, E. & De Strooper, B. The amyloid hypothesis in Alzheimer disease: New insights from new therapeutics. *Nat. Rev. Drug Discov.* **21**, 306–318. 10.1038/s41573-022-00391-w (2022). 35177833 10.1038/s41573-022-00391-w

[2] Jack, C. R. Jr. et al. Tracking pathophysiological processes in Alzheimer’s disease: An updated hypothetical model of dynamic biomarkers. *Lancet Neurol.* **12**, 207–216. 10.1016/S1474-4422(12)70291-0 (2013). 23332364 10.1016/S1474-4422(12)70291-0 PMC3622225

[3] Panza, F., Lozupone, M., Logroscino, G. & Imbimbo, B. P. A critical appraisal of amyloid-beta-targeting therapies for Alzheimer disease. *Nat. Rev. Neurol.* **15**, 73–88. 10.1038/s41582-018-0116-6 (2019). 30610216 10.1038/s41582-018-0116-6

[4] van Dyck, C. H. et al. Lecanemab in early Alzheimer’s disease. *N. Engl. J. Med.* **388**, 9–21. 10.1056/NEJMoa2212948 (2023). 36449413 10.1056/NEJMoa2212948

[5] Budd Haeberlein, S. et al. Two randomized phase 3 studies of aducanumab in early Alzheimer’s disease. *J. Prev. Alzheimers Dis.* **9**, 197–210. 10.14283/jpad.2022.30 (2022). 35542991 10.14283/jpad.2022.30

[6] Sims, J. R. et al. Donanemab in early symptomatic alzheimer disease: The TRAILBLAZER-ALZ 2 randomized clinical trial. *JAMA* **330**, 512–527. 10.1001/jama.2023.13239 (2023). 37459141 10.1001/jama.2023.13239 PMC10352931

[7] Lancet. Lecanemab for Alzheimer’s disease: tempering hype and hope. *Lancet* **400**, 1899. 10.1016/S0140-6736(22)02480-1 (2022). 10.1016/S0140-6736(22)02480-1 36463893

[8] Thambisetty, M. & Howard, R. Lecanemab trial in AD brings hope but requires greater clarity. *Nat. Rev. Neurol.* **19**, 132–133. 10.1038/s41582-022-00768-w (2023). 36609712 10.1038/s41582-022-00768-w

[9] Knopman, D. S., Jones, D. T. & Greicius, M. D. Failure to demonstrate efficacy of aducanumab: An analysis of the EMERGE and ENGAGE trials as reported by Biogen, December 2019. *Alzheimers Dement* **17**, 696–701. 10.1002/alz.12213 (2021). 33135381 10.1002/alz.12213

[10] Sturchio, A. et al. High soluble amyloid-beta42 predicts normal cognition in amyloid-positive individuals with Alzheimer’s disease-causing mutations. *J. Alzheimers Dis.* **90**, 333–348. 10.3233/JAD-220808 (2022). 36120786 10.3233/JAD-220808 PMC9661329

[11] Page, M. J. et al. The PRISMA 2020 statement: An updated guideline for reporting systematic reviews. *BMJ* **372**, n71. 10.1136/bmj.n71 (2021). 33782057 10.1136/bmj.n71 PMC8005924

[12] Sterne, J. A. C. et al. RoB 2: A revised tool for assessing risk of bias in randomised trials. *BMJ* **366**, l4898. 10.1136/bmj.l4898 (2019). 31462531 10.1136/bmj.l4898

[13] Guyatt, G. H. et al. GRADE: An emerging consensus on rating quality of evidence and strength of recommendations. *BMJ* **336**, 924–926. 10.1136/bmj.39489.470347.AD (2008). 18436948 10.1136/bmj.39489.470347.AD PMC2335261

[14] Egger, M., Davey Smith, G., Schneider, M. & Minder, C. Bias in meta-analysis detected by a simple, graphical test. *BMJ* **315**, 629–634. 10.1136/bmj.315.7109.629 (1997). 9310563 10.1136/bmj.315.7109.629 PMC2127453

[15] Higgins, J. P. T., Li, T. & Deeks, J. J. (editors). Chapter 6: Choosing effect measures and computing estimates of effect. In: Higgins JPT, Thomas J, Chandler J, Cumpston M, Li T, Page MJ, Welch VA (editors). Cochrane Handbook for Systematic Reviews of Interventions version 6.3 (updated February 2022). Cochrane, 2022. Available from www.training.cochrane.org/handbook.

[16] Viechtbauer, W. Bias and efficiency of meta-analytic variance estimators in the random-effects model. *J. Educ. Behav. Stat.* **30**, 261–293. 10.3102/10769986030003261 (2005).

[17] Knapp, G. & Hartung, J. Improved tests for a random effects meta-regression with a single covariate. *Stat. Med.* **22**, 2693–2710. 10.1002/sim.1482 (2003). 12939780 10.1002/sim.1482

[18] Mantel, N. & Haenszel, W. Statistical aspects of the analysis of data from retrospective studies of disease. *J. Natl. Cancer Inst.* **22**, 719–748 (1959). 13655060

[19] Schünemann, H. J., Vist, G. E., Higgins, J. P. T., Santesso, N., Deeks, J. J., Glasziou, P., Akl, E. A., Guyatt, G. H. Chapter 15: Interpreting results and drawing conclusions. In: Higgins, J. P. T., Thomas, J., Chandler, J., Cumpston, M., Li, T., Page, M. J., Welch VA (editors). Cochrane Handbook for Systematic Reviews of Interventions version 6.3 (updated February 2022). Cochrane, 2022. Available from www.training.cochrane.org/handbook.

[20] Kraemer, H. C. & Kupfer, D. J. Size of treatment effects and their importance to clinical research and practice. *Biol. Psychiatry* **59**, 990–996. 10.1016/j.biopsych.2005.09.014 (2006). 16368078 10.1016/j.biopsych.2005.09.014

[21] Soderberg, L. et al. Lecanemab, aducanumab, and gantenerumab - binding profiles to different forms of amyloid-beta might explain efficacy and side effects in clinical trials for alzheimer’s disease. *Neurotherapeutics*10.1007/s13311-022-01308-6 (2022). 36253511 10.1007/s13311-022-01308-6 PMC10119362

[22] Doody, R. S. et al. Phase 3 trials of solanezumab for mild-to-moderate Alzheimer’s disease. *N. Engl. J. Med.* **370**, 311–321. 10.1056/NEJMoa1312889 (2014). 24450890 10.1056/NEJMoa1312889

[23] Honig, L. S. et al. Trial of solanezumab for mild dementia due to Alzheimer’s disease. *N. Engl. J. Med.* **378**, 321–330. 10.1056/NEJMoa1705971 (2018). 29365294 10.1056/NEJMoa1705971

[24] Sperling, R. A. et al. Trial of solanezumab in preclinical alzheimer’s disease. *N. Engl. J. Med.*10.1056/NEJMoa2305032 (2023). 37458272 10.1056/NEJMoa2305032 PMC10559996

[25] Salloway, S. et al. Two phase 3 trials of bapineuzumab in mild-to-moderate Alzheimer’s disease. *N. Engl. J. Med.* **370**, 322–333. 10.1056/NEJMoa1304839 (2014). 24450891 10.1056/NEJMoa1304839 PMC4159618

[26] Vandenberghe, R. et al. Bapineuzumab for mild to moderate Alzheimer’s disease in two global, randomized, phase 3 trials. *Alzheimers Res. Ther.* **8**, 18. 10.1186/s13195-016-0189-7 (2016). 27176461 10.1186/s13195-016-0189-7 PMC4866415

[27] Ostrowitzki, S. et al. Evaluating the safety and efficacy of crenezumab vs placebo in adults with early alzheimer disease: Two phase 3 randomized placebo-controlled trials. *JAMA Neurol.* **79**, 1113–1121. 10.1001/jamaneurol.2022.2909 (2022). 36121669 10.1001/jamaneurol.2022.2909 PMC9486635

[28] Ostrowitzki, S. et al. A phase III randomized trial of gantenerumab in prodromal Alzheimer’s disease. *Alzheimers Res. Ther.* **9**, 95. 10.1186/s13195-017-0318-y (2017). 29221491 10.1186/s13195-017-0318-y PMC5723032

[29] Voyle, N. et al. THE effect of low doses of gantenerumab on amyloid and tau biomarkers in cerebrospinal fluid (CSF) in the marguerite road study. *Alzheimer’s Dementia* **14**, P240. 10.1016/j.jalz.2018.06.2379 (2018).

[30] Bateman, R. J. et al. Two phase 3 trials of gantenerumab in early alzheimer’s disease. *N. Engl. J. Med.* **389**, 1862–1876. 10.1056/NEJMoa2304430 (2023). 37966285 10.1056/NEJMoa2304430 PMC10794000

[31] Salloway S., Lee E., Papka M., et al. TRAILBLAZER-ALZ 4: Topline study results directly comparing donanemab to aducanumab on amyloid lowering in early, symptomatic Alzheimer’s disease. In *Clinical Trials on Alzheimer’s Disease (CTAD)—15th Annual Conference*, 2022. Available from https://medically.gene.com.

[32] Lansdall, C. J. et al. Establishing clinically meaningful change on outcome assessments frequently used in trials of mild cognitive impairment due to Alzheimer’s disease. *J. Prev. Alzheimers Dis.* **10**, 9–18. 10.14283/jpad.2022.102 (2023). 36641605 10.14283/jpad.2022.102

[33] Avgerinos, K. I., Ferrucci, L. & Kapogiannis, D. Effects of monoclonal antibodies against amyloid-beta on clinical and biomarker outcomes and adverse event risks: A systematic review and meta-analysis of phase III RCTs in Alzheimer’s disease. *Ageing Res. Rev.* **68**, 101339. 10.1016/j.arr.2021.101339 (2021). 33831607 10.1016/j.arr.2021.101339 PMC8161699

[34] Lu, L. et al. Anti-Abeta agents for mild to moderate Alzheimer’s disease: systematic review and meta-analysis. *J. Neurol. Neurosurg. Psychiatry* **91**, 1316–1324. 10.1136/jnnp-2020-323497 (2020). 33046560 10.1136/jnnp-2020-323497

[35] Petersen, R. C. et al. Expectations and clinical meaningfulness of randomized controlled trials. *Alzheimers Dement*10.1002/alz.12959 (2023). 36748826 10.1002/alz.12959 PMC11156248

