# Supplementary material for: Critical assessment of anti-amyloid-β monoclonal antibodies effects in Alzheimer’s disease: a systematic review and meta-analysis highlighting target engagement and clinical meaningfulness
Source: Sci Rep. 2024 Oct 28;14:25741. doi: 10.1038/s41598-024-75204-8 (PMC11520896; doi:10.1038/s41598-024-75204-8)
Supplement: Supplementary file 1 — Supplementary Material 1 [file 41598_2024_75204_MOESM1_ESM.pdf]

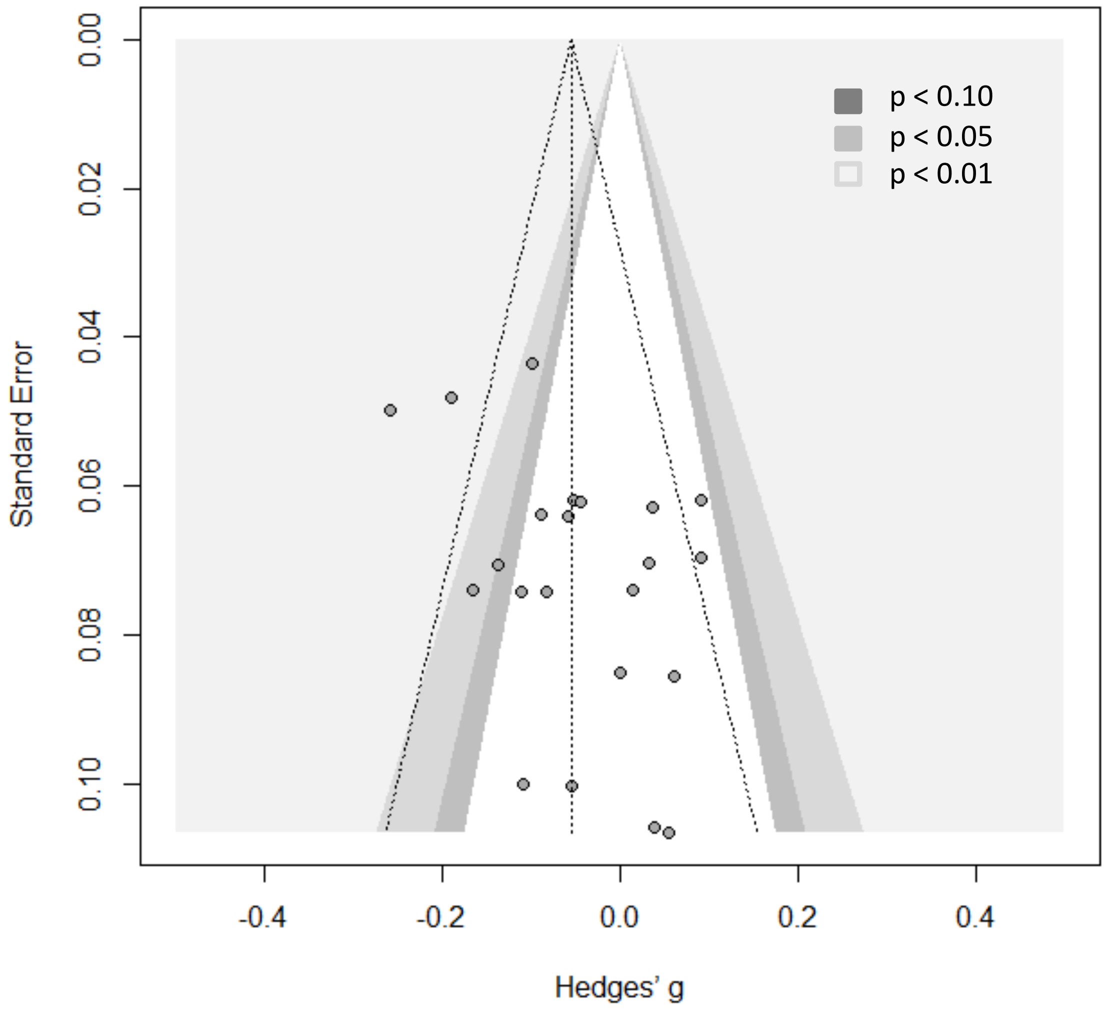

Figure S1. Funnel Plot for assessment of publications bias based on primary outcome (CDR-SB) effect sizes

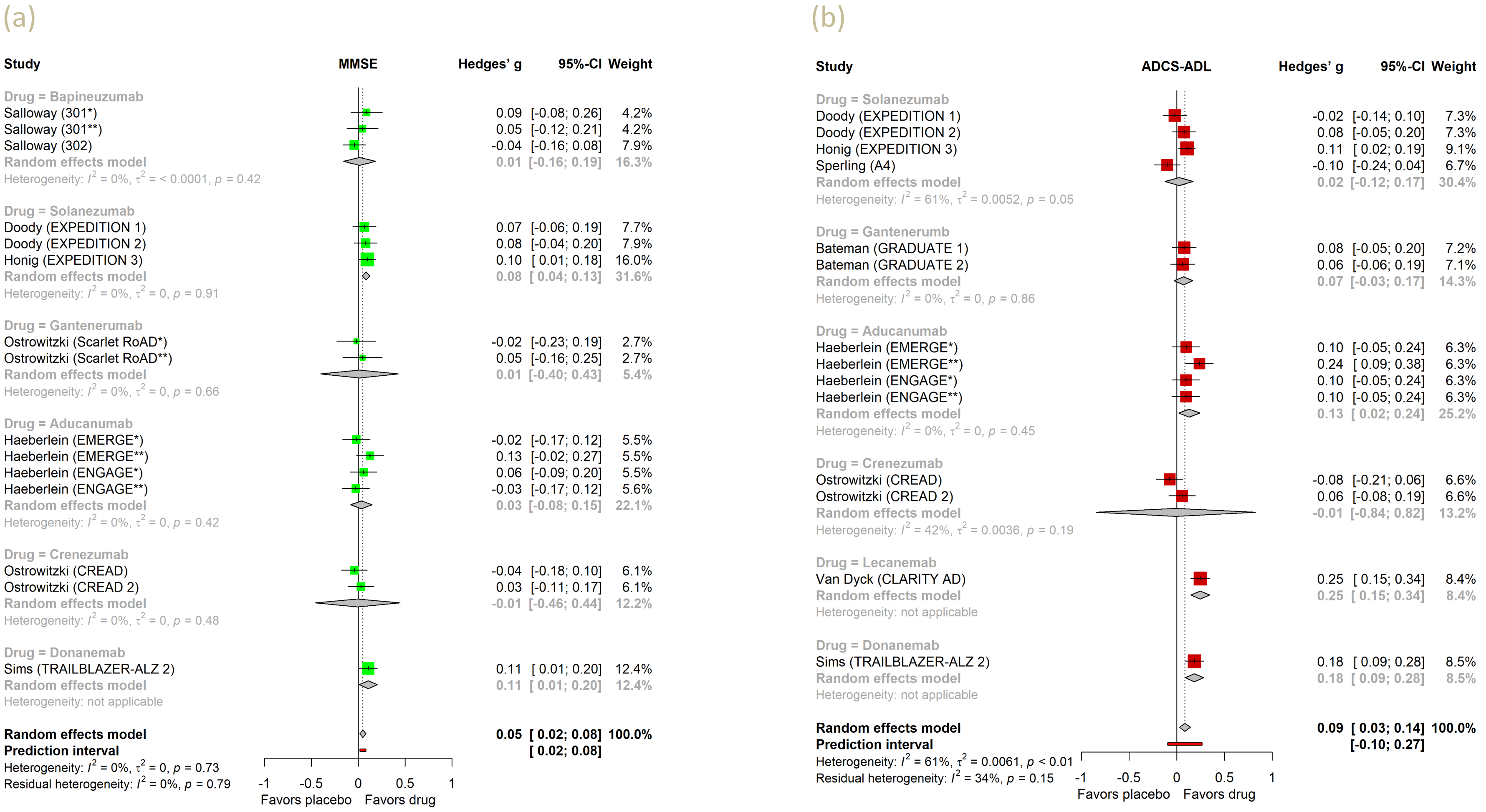

Figure S2. Forest plots of (a) Mini Mental State Examination (MMSE) and (b) Alzheimer's Disease Cooperative Study - Activities of Daily Living (ADCS-ADL) meta-analyses with subgroup analyses by drug

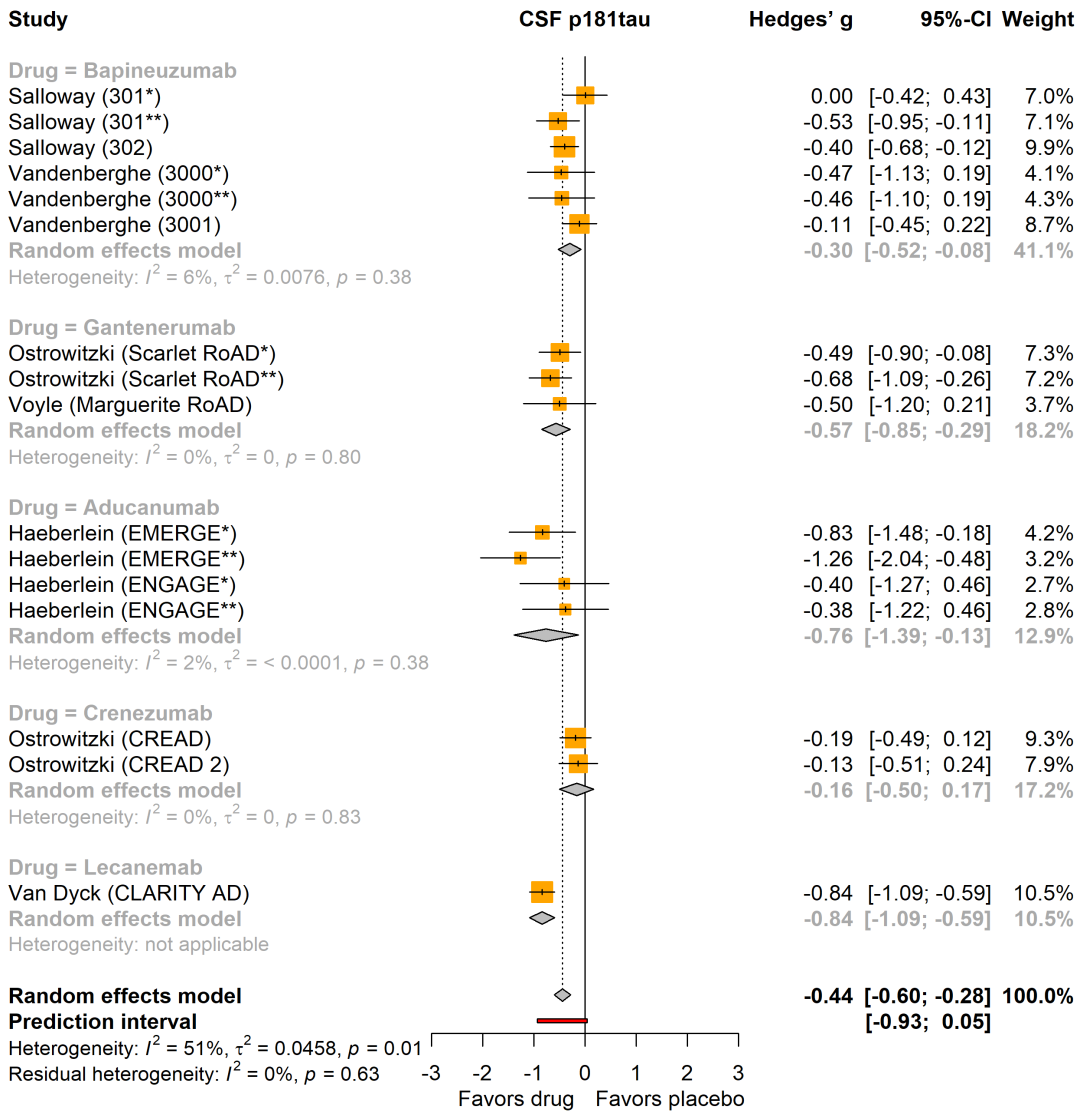

Figure S3. Forest plot of CSF p181-tau meta-analysis with subgroup analysis by drug

Study or Subgroup	mAbs Mean	mAbs SD	mAbs Total	Placebo Mean	Placebo SD	Placebo Total	Weight	Std. Mean Difference IV, Random, 95% CI
**1.6.1 Aducanumab**								
Haeberlein (EMERGE*)	148.48	127.64	33	−30.34	127.21	14	7.5%	1.38 [0.69, 2.07]
Haeberlein (EMERGE**)	287.52	121.76	17	−30.34	127.21	14	6.5%	2.49 [1.52, 3.46]
Haeberlein (ENGAGE*)	95.76	154.65	19	8.55	144.81	8	7.0%	0.56 [−0.29, 1.40]
Haeberlein (ENGAGE**)	206.87	157.5	17	8.55	144.81	7	6.6%	1.24 [0.28, 2.20]
**Subtotal (95% CI)**			**86**			**43**	**27.7%**	**1.39 [0.66, 2.13]**
Heterogeneity: $Tau^2 = 0.37$; $Chi^2 = 8.79$, df = 3 (P = 0.03); $I^2 = 66\%$								
Test for overall effect: Z = 3.72 (P = 0.0002)								
**1.6.2 Crenezumab**								
Ostrowitzki (CREAD)	294.4	139.58	17	16.16	155.64	23	7.3%	1.83 [1.07, 2.58]
Ostrowitzki (CREAD 2)	321.94	129.69	33	13.67	135.01	26	7.6%	2.30 [1.63, 2.97]
**Subtotal (95% CI)**			**50**			**49**	**14.9%**	**2.09 [1.59, 2.60]**
Heterogeneity: $Tau^2 = 0.00$; $Chi^2 = 0.85$, df = 1 (P = 0.36); $I^2 = 0\%$								
Test for overall effect: Z = 8.19 (P < 0.00001)								
**1.6.3 Gantenerumab**								
Ostrowitzki (Scarlet RoAD*)	11.64	34.94	64	23.76	61.58	35	8.4%	−0.26 [−0.67, 0.15]
Ostrowitzki (Scarlet RoAD**)	77.79	77.8	65	23.76	61.58	34	8.3%	0.74 [0.31, 1.17]
Marguerite RoAD	32.34	117.61	12	−19.9	74.37	23	7.5%	0.56 [−0.15, 1.27]
**Subtotal (95% CI)**			**141**			**92**	**24.1%**	**0.33 [−0.36, 1.02]**
Heterogeneity: $Tau^2 = 0.30$; $Chi^2 = 11.56$, df = 2 (P = 0.003); $I^2 = 83\%$								
Test for overall effect: Z = 0.93 (P = 0.35)								
**1.6.4 Lecanemab**								
Van Dyck (CLARITY AD)	281.22	282.5	101	−5.95	271.83	97	8.6%	1.03 [0.73, 1.33]
**Subtotal (95% CI)**			**101**			**97**	**8.6%**	**1.03 [0.73, 1.33]**
Heterogeneity: Not applicable								
Test for overall effect: Z = 6.81 (P < 0.00001)								
**1.6.5 Solanezumab**								
Doody (EXPEDITION 1)	471.4	1,938.83	20	−242.3	2,185.31	25	7.9%	0.34 [−0.26, 0.93]
Doody (EXPEDITION 2)	726.6	780.36	44	323.8	659.15	32	8.2%	0.54 [0.08, 1.01]
Honig (EXPEDITION 3)	510.29	302.29	131	−46.72	199.28	127	8.6%	2.16 [1.85, 2.47]
**Subtotal (95% CI)**			**195**			**184**	**24.7%**	**1.03 [−0.23, 2.29]**
Heterogeneity: $Tau^2 = 1.19$; $Chi^2 = 48.73$, df = 2 (P < 0.00001); $I^2 = 96\%$								
Test for overall effect: Z = 1.60 (P = 0.11)								
**Total (95% CI)**			**573**			**465**	**100.0%**	**1.12 [0.64, 1.61]**
Heterogeneity: $Tau^2 = 0.68$; $Chi^2 = 130.50$, df = 12 (P < 0.00001); $I^2 = 91\%$								
Test for overall effect: Z = 4.57 (P < 0.00001)								
Test for subgroup differences: $Chi^2 = 19.87$, df = 4 (P = 0.0005), $I^2 = 79.9\%$								

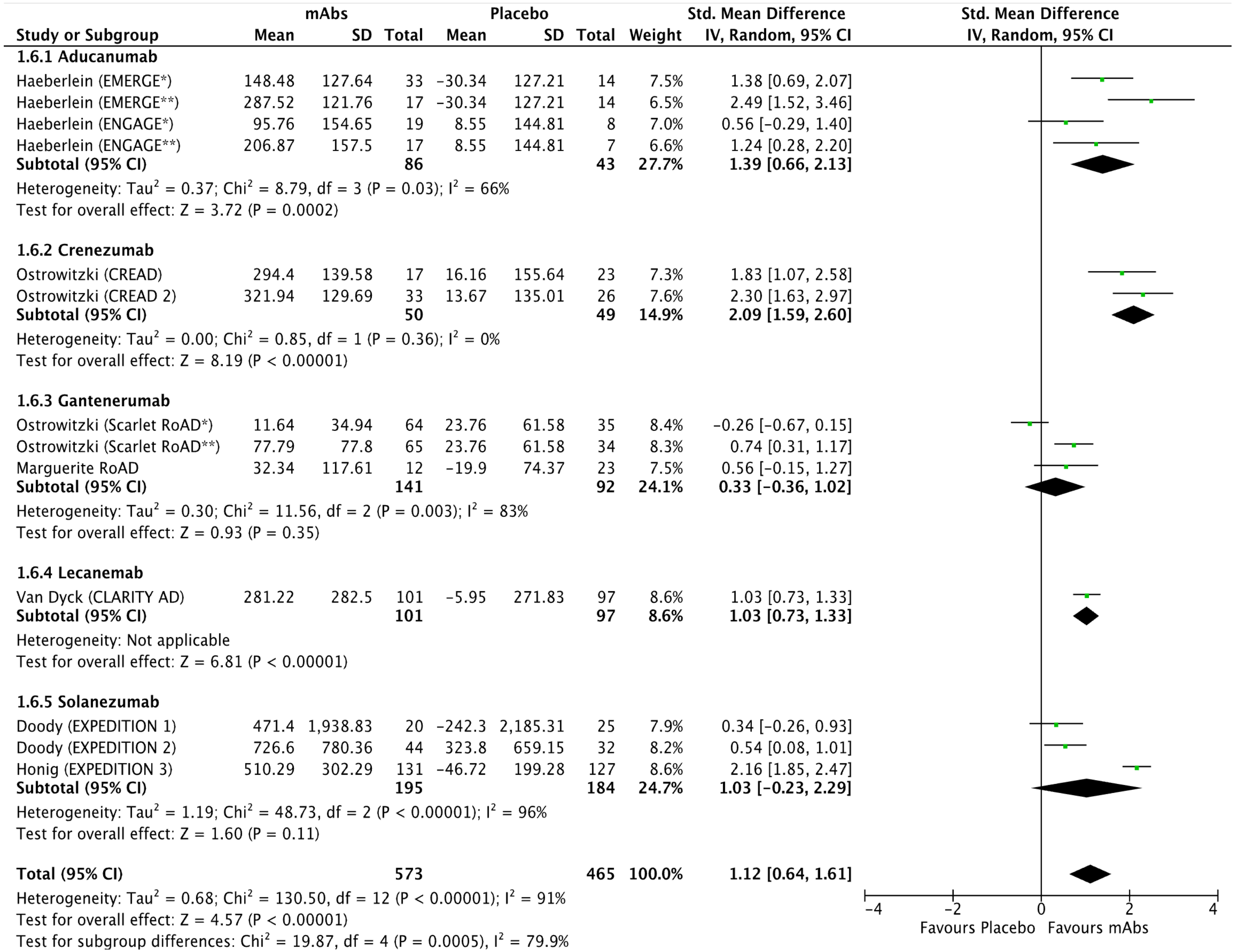

Figure S4. Forest plot of CSF $A\beta_{42}$ meta-analysis with subgroup analysis by drug

Study or Subgroup	mAbs Mean	mAbs SD	mAbs Total	Placebo Mean	Placebo SD	Placebo Total	Weight	Std. Mean Difference IV, Random, 95% CI
**1.9.1 Crenezumab**								
Ostrowitzki (CREAD)	6,019.13	3,072.9	17	−99.19	3,339.18	22	12.6%	1.86 [1.09, 2.63]
Ostrowitzki (CREAD 2)	6,390.57	3,394.18	31	360	3,698.11	25	13.8%	1.68 [1.07, 2.30]
**Subtotal (95% CI)**			**48**			**47**	**26.4%**	**1.75 [1.27, 2.23]**
Heterogeneity: $Tau^2 = 0.00$; $Chi^2 = 0.12$, df = 1 (P = 0.73); $I^2 = 0\%$								
Test for overall effect: Z = 7.13 (P < 0.00001)								
**1.9.2 Gantenerumab**								
Marguerite RoAD	−1,361.58	1,464.93	12	−1,258	2,520.32	23	13.1%	−0.05 [−0.74, 0.65]
**Subtotal (95% CI)**			**12**			**23**	**13.1%**	**−0.05 [−0.74, 0.65]**
Heterogeneity: Not applicable								
Test for overall effect: Z = 0.13 (P = 0.90)								
**1.9.3 Lecanemab**								
Van Dyck (CLARITY AD)	−407.69	2,930.19	71	−93.64	2,844.33	71	15.7%	−0.11 [−0.44, 0.22]
**Subtotal (95% CI)**			**71**			**71**	**15.7%**	**−0.11 [−0.44, 0.22]**
Heterogeneity: Not applicable								
Test for overall effect: Z = 0.64 (P = 0.52)								
**1.9.4 Solanezumab**								
Doody (EXPEDITION 1)	1,325.4	9,588.27	20	−1,902.1	11,526.61	25	14.0%	0.30 [−0.30, 0.89]
Doody (EXPEDITION 2)	2,156.8	11,080.1	44	−876.4	9,613.82	32	14.9%	0.29 [−0.17, 0.74]
Honig (EXPEDITION 3)	3,531.06	4,331.08	131	−993.4	3,833.71	126	16.0%	1.10 [0.84, 1.36]
**Subtotal (95% CI)**			**195**			**183**	**44.8%**	**0.60 [−0.02, 1.21]**
Heterogeneity: $Tau^2 = 0.25$; $Chi^2 = 12.65$, df = 2 (P = 0.002); $I^2 = 84\%$								
Test for overall effect: Z = 1.89 (P = 0.06)								
**Total (95% CI)**			**326**			**324**	**100.0%**	**0.70 [0.15, 1.26]**
Heterogeneity: $Tau^2 = 0.49$; $Chi^2 = 59.58$, df = 6 (P < 0.00001); $I^2 = 90\%$								
Test for overall effect: Z = 2.48 (P = 0.01)								
Test for subgroup differences: $Chi^2 = 41.26$, df = 3 (P < 0.00001), $I^2 = 92.7\%$								

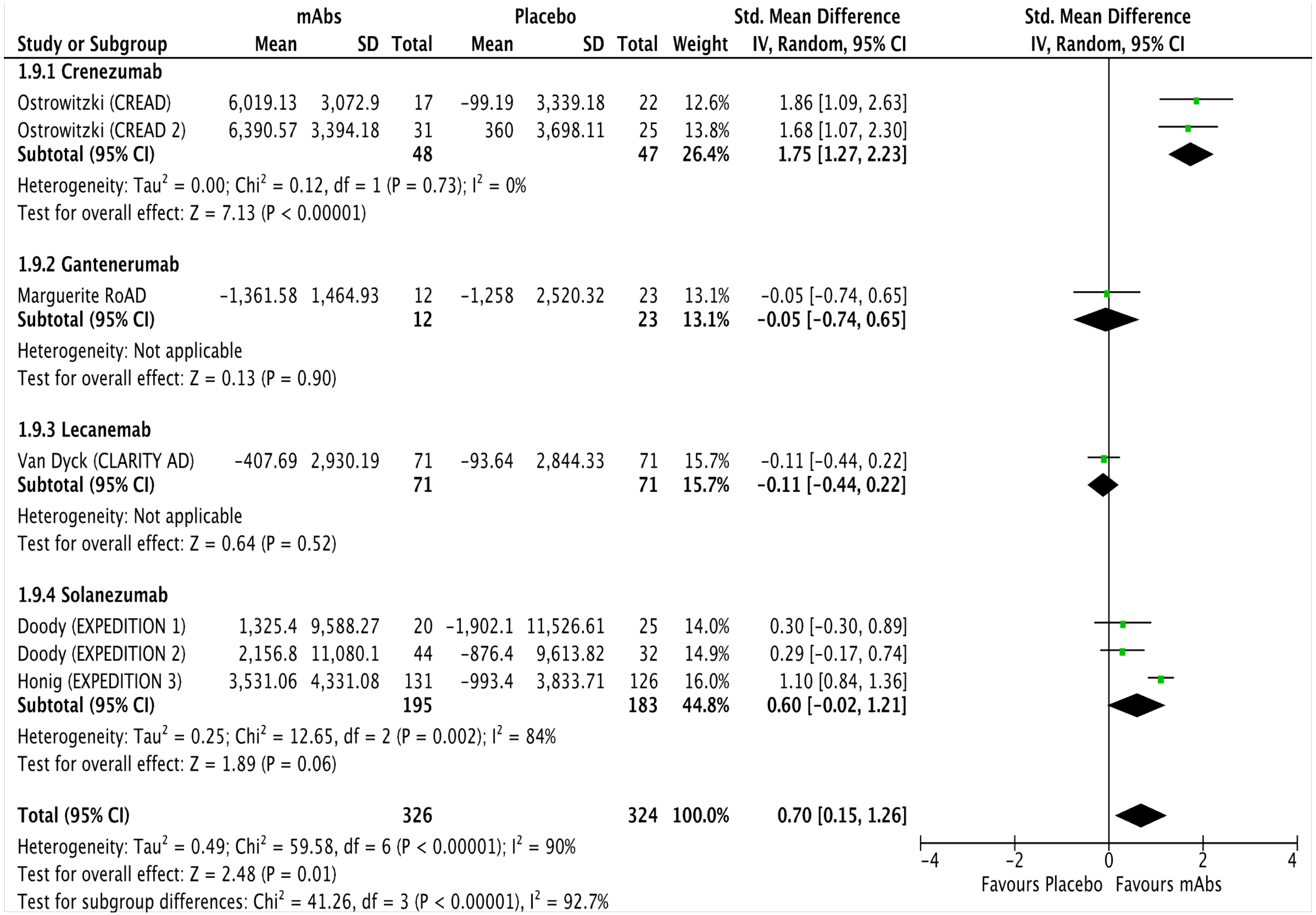

Figure S5. Forest plot of CSF $A\beta_{40}$ meta-analysis with subgroup analysis by drug

Study or Subgroup	mAbs Events	mAbs Total	Placebo Events	Placebo Total	Weight	Risk Ratio M-H, Random, 95% CI
**1.3.1 Aducanumab**						
Haeberlein (EMERGE*)	0	544	3	274	1.0%	0.07 [0.00, 1.39]
Haeberlein (EMERGE**)	6	547	2	273	3.1%	1.50 [0.30, 7.37]
Haeberlein (ENGAGE*)	3	549	0	270	1.0%	3.45 [0.18, 66.53]
Haeberlein (ENGAGE**)	2	558	0	270	0.9%	2.42 [0.12, 50.31]
**Subtotal (95% CI)**		**2198**		**1087**	**6.0%**	**1.07 [0.24, 4.80]**
Total events	11		5			
Heterogeneity: Tau^2 = 0.74; Chi^2 = 4.32, df = 3 (P = 0.23); I^2 = 31%						
Test for overall effect: Z = 0.08 (P = 0.93)						
**1.3.2 Bapineuzumab**						
Salloway (301*)	4	337	4	262	4.0%	0.78 [0.20, 3.08]
Salloway (301**)	7	329	3	262	4.2%	1.86 [0.49, 7.12]
Salloway (302)	15	673	5	448	6.7%	2.00 [0.73, 5.46]
Vandenberghe (3000*)	1	267	2	172	1.5%	0.32 [0.03, 3.53]
Vandenberghe (3000**)	1	263	1	172	1.1%	0.65 [0.04, 10.39]
Vandenberghe (3001)	4	654	4	439	4.0%	0.67 [0.17, 2.67]
**Subtotal (95% CI)**		**2523**		**1755**	**21.6%**	**1.16 [0.65, 2.09]**
Total events	32		19			
Heterogeneity: Tau^2 = 0.00; Chi^2 = 3.80, df = 5 (P = 0.58); I^2 = 0%						
Test for overall effect: Z = 0.50 (P = 0.62)						
**1.3.3 Donanemab**						
Sims (TRAILBLAZER-ALZ 2)	16	853	10	874	9.5%	1.64 [0.75, 3.59]
**Subtotal (95% CI)**		**853**		**874**	**9.5%**	**1.64 [0.75, 3.59]**
Total events	16		10			
Heterogeneity: Not applicable						
Test for overall effect: Z = 1.24 (P = 0.22)						
**1.3.4 Crenezumab**						
Ostrowitzki (CREAD)	8	404	5	405	5.8%	1.60 [0.53, 4.86]
Ostrowitzki (CREAD 2)	0	404	6	398	1.0%	0.08 [0.00, 1.34]
**Subtotal (95% CI)**		**808**		**803**	**6.8%**	**0.45 [0.02, 10.52]**
Total events	8		11			
Heterogeneity: Tau^2 = 4.07; Chi^2 = 4.30, df = 1 (P = 0.04); I^2 = 77%						
Test for overall effect: Z = 0.49 (P = 0.62)						
**1.3.5 Gantenerumab**						
Ostrowitzki (Scarlet RoAD*)	0	271	3	133	1.0%	0.07 [0.00, 1.35]
Ostrowitzki (Scarlet RoAD**)	2	260	3	133	2.6%	0.34 [0.06, 2.02]
Bateman (GRADUATE 1)	3	503	10	481	4.5%	0.29 [0.08, 1.04]
Bateman (GRADUATE 2)	7	501	5	474	5.5%	1.32 [0.42, 4.14]
Marguerite RoAD	8	192	11	195	8.0%	0.74 [0.30, 1.80]
**Subtotal (95% CI)**		**1727**		**1416**	**21.6%**	**0.55 [0.26, 1.15]**
Total events	20		32			
Heterogeneity: Tau^2 = 0.21; Chi^2 = 5.80, df = 4 (P = 0.21); I^2 = 31%						
Test for overall effect: Z = 1.60 (P = 0.11)						
**1.3.6 Lecanemab**						
Van Dyck (CLARITY AD)	6	898	7	897	5.9%	0.86 [0.29, 2.54]
**Subtotal (95% CI)**		**898**		**897**	**5.9%**	**0.86 [0.29, 2.54]**
Total events	6		7			
Heterogeneity: Not applicable						
Test for overall effect: Z = 0.28 (P = 0.78)						
**1.3.7 Solanezumab**						
Doody (EXPEDITION 1/2)	24	1027	19	1025	13.1%	1.26 [0.69, 2.29]
Honig (EXPEDITION 3)	9	1054	17	1067	9.2%	0.54 [0.24, 1.20]
Sperling (A4)	6	572	8	591	6.2%	0.77 [0.27, 2.22]
**Subtotal (95% CI)**		**2653**		**2683**	**28.6%**	**0.86 [0.50, 1.49]**
Total events	39		44			
Heterogeneity: Tau^2 = 0.07; Chi^2 = 2.91, df = 2 (P = 0.23); I^2 = 31%						
Test for overall effect: Z = 0.53 (P = 0.60)						
**Total (95% CI)**		**11660**		**9515**	**100.0%**	**0.91 [0.68, 1.23]**
Total events	132		128			
Heterogeneity: Tau^2 = 0.09; Chi^2 = 25.64, df = 21 (P = 0.22); I^2 = 18%						
Test for overall effect: Z = 0.61 (P = 0.55)						
Test for subgroup differences: Chi^2 = 4.83, df = 6 (P = 0.57), I^2 = 0%						

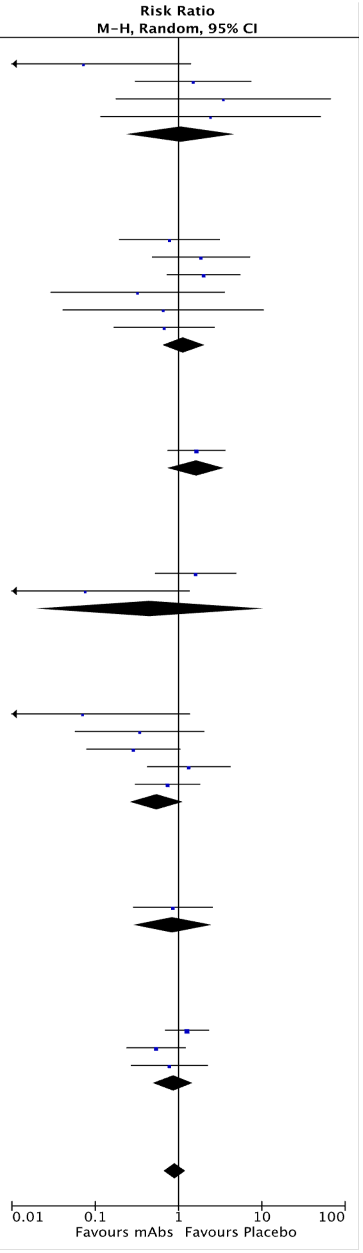

Figure S6. Forest plot of all-cause mortality meta-analysis with subgroup analysis by drug

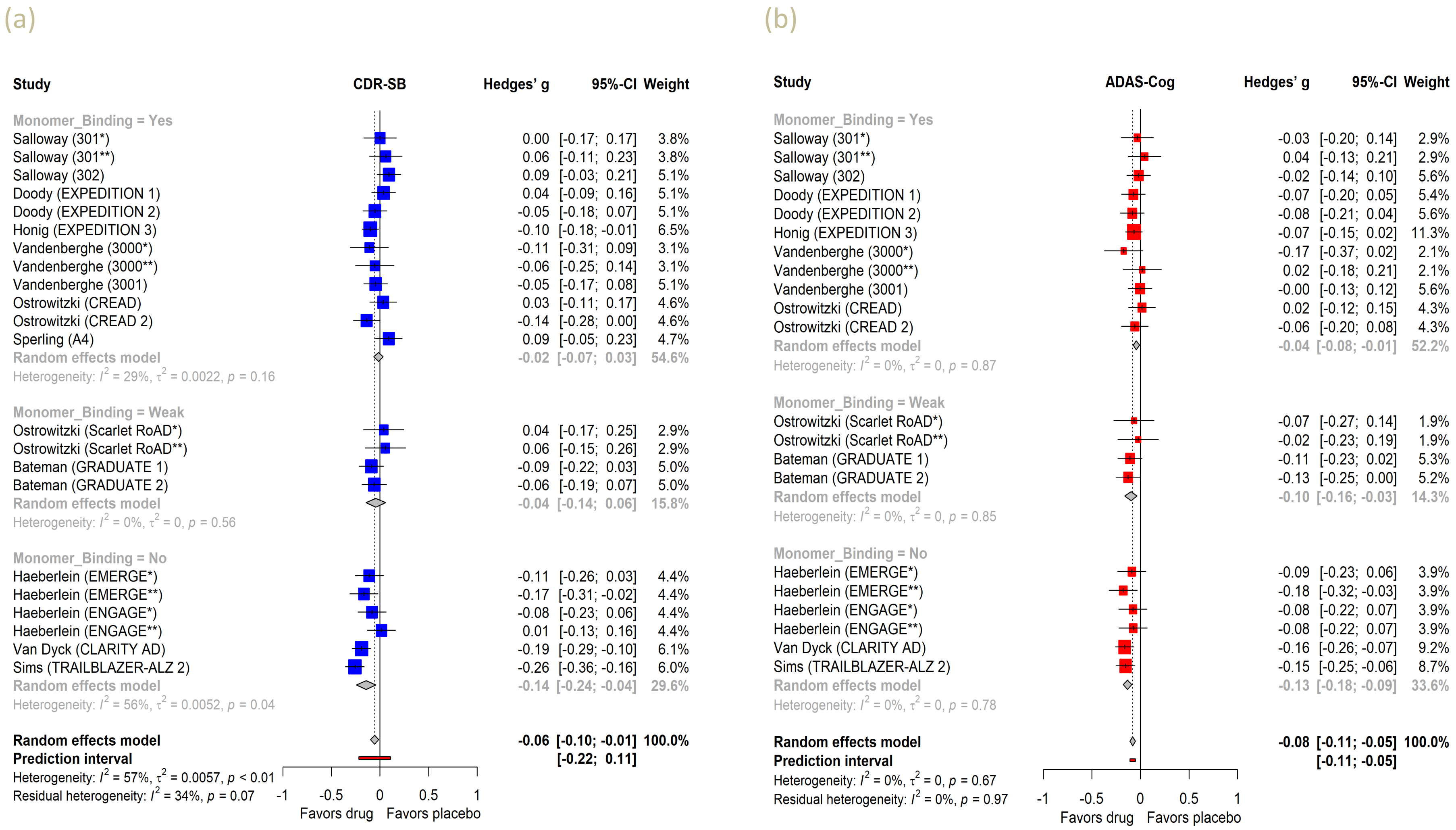

Figure S7. Forest plots of (a) Clinical Dementia Rating Scale Sum of Boxes (CDR-SB) and (b) AD Assessment Scale–Cognitive Subscale (ADAS-Cog) meta-analyses with with subgroup analyses by binding affinity to Aβ monomers

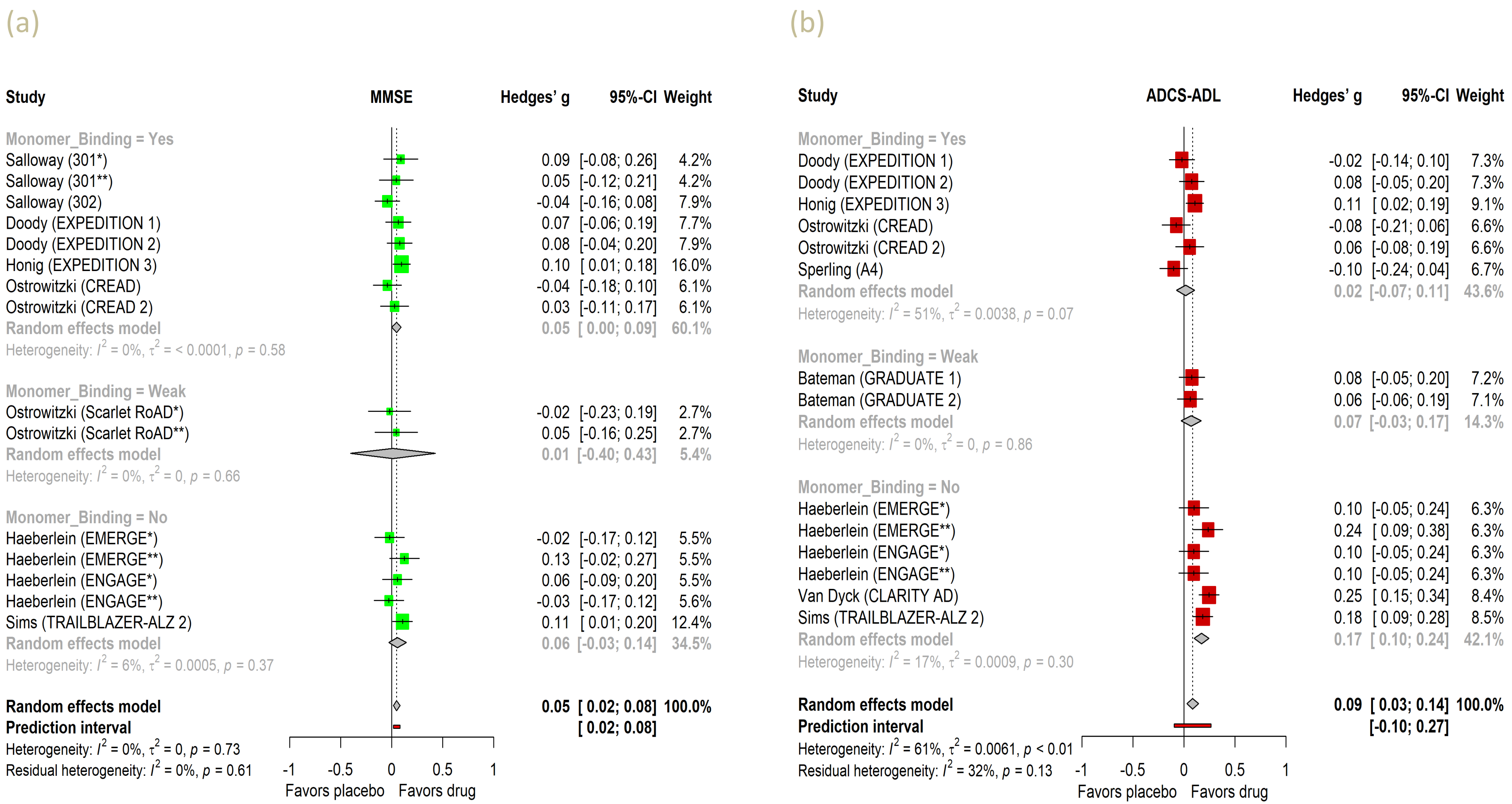

Figure S8. Forest plots of (a) Mini Mental State Examination (MMSE) and (b) Alzheimer's Disease Cooperative Study - Activities of Daily Living (ADCS-ADL) meta-analyses with subgroup analyses by binding affinity to Aβ monomers

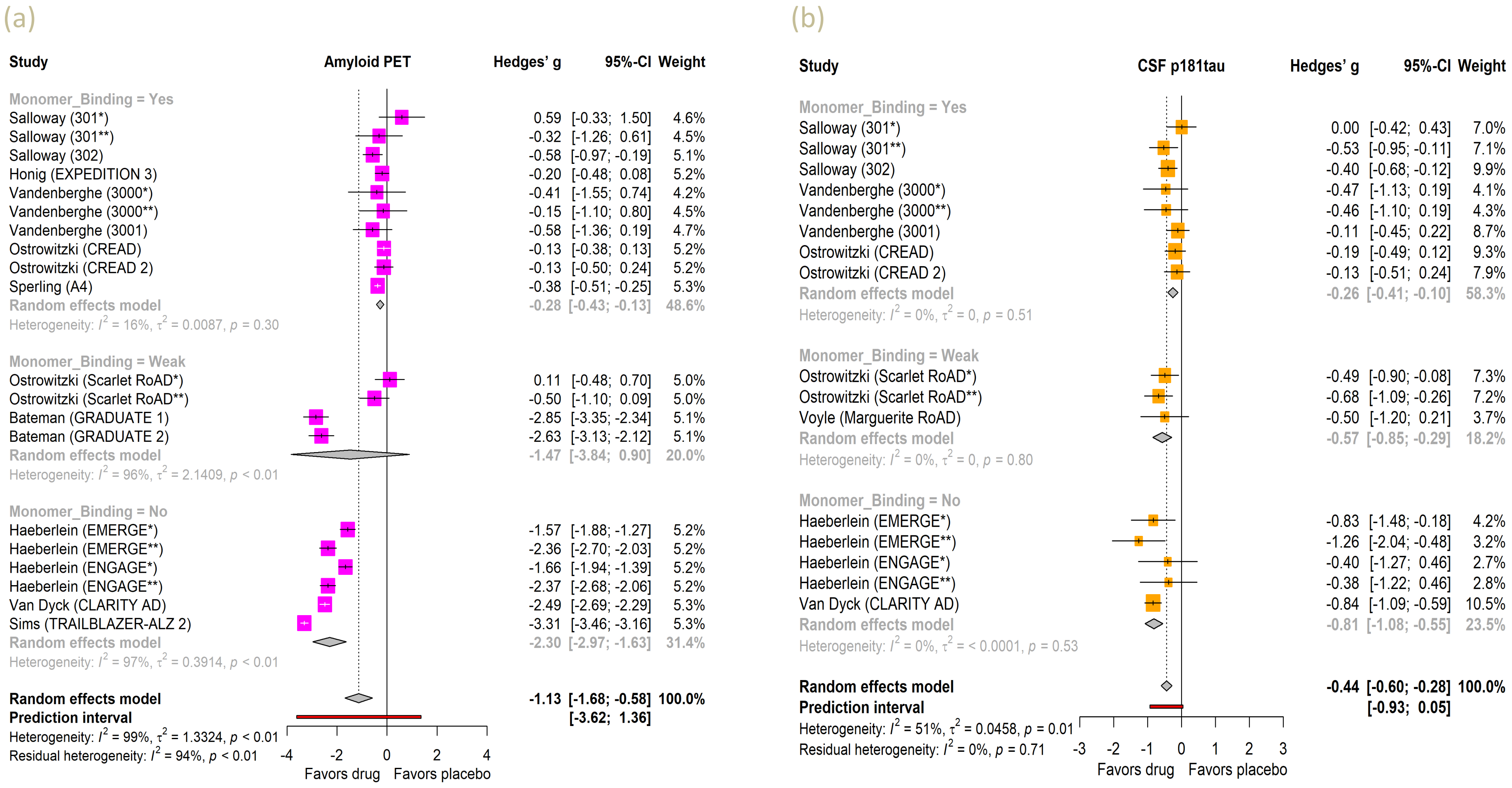

Figure S9. Forest plots of (a) Amyloid PET and (b) CSF p181-tau meta-analyses with subgroup analyses by binding affinity to Aβ monomers

Study or Subgroup	mAbs Mean	mAbs SD	mAbs Total	Placebo Mean	Placebo SD	Placebo Total	Weight	Std. Mean Difference IV, Random, 95% CI
**1.5.1 Monomer Binding = Yes**								
Doody (EXPEDITION 1)	471.4	1,938.83	20	−242.3	2,185.31	25	7.9%	0.34 [−0.26, 0.93]
Doody (EXPEDITION 2)	726.6	780.36	44	323.8	659.15	32	8.2%	0.54 [0.08, 1.01]
Honig (EXPEDITION 3)	510.29	302.29	131	−46.72	199.28	127	8.6%	2.16 [1.85, 2.47]
Ostrowitzki (CREAD)	294.4	139.58	17	16.16	155.64	23	7.3%	1.83 [1.07, 2.58]
Ostrowitzki (CREAD 2)	321.94	129.69	33	13.67	135.01	26	7.6%	2.30 [1.63, 2.97]
**Subtotal (95% CI)**			**245**			**233**	**39.6%**	**1.43 [0.58, 2.29]**
Heterogeneity: $Tau^2 = 0.86$; $Chi^2 = 54.66$, df = 4 ($P < 0.00001$); $I^2 = 93\%$								
Test for overall effect: Z = 3.28 (P = 0.001)								
**1.5.2 Monomer Binding = Weak**								
Ostrowitzki (Scarlet RoAD*)	11.64	34.94	64	23.76	61.58	35	8.4%	−0.26 [−0.67, 0.15]
Ostrowitzki (Scarlet RoAD**)	77.79	77.8	65	23.76	61.58	34	8.3%	0.74 [0.31, 1.17]
Marguerite RoAD	32.34	117.61	12	−19.9	74.37	23	7.5%	0.56 [−0.15, 1.27]
**Subtotal (95% CI)**			**141**			**92**	**24.1%**	**0.33 [−0.36, 1.02]**
Heterogeneity: $Tau^2 = 0.30$; $Chi^2 = 11.56$, df = 2 ($P = 0.003$); $I^2 = 83\%$								
Test for overall effect: Z = 0.93 (P = 0.35)								
**1.5.3 Monomer Binding = No**								
Haeberlein (EMERGE*)	148.48	127.64	33	−30.34	127.21	14	7.5%	1.38 [0.69, 2.07]
Haeberlein (EMERGE**)	287.52	121.76	17	−30.34	127.21	14	6.5%	2.49 [1.52, 3.46]
Haeberlein (ENGAGE*)	95.76	154.65	19	8.55	144.81	8	7.0%	0.56 [−0.29, 1.40]
Haeberlein (ENGAGE**)	206.87	157.5	17	8.55	144.81	7	6.6%	1.24 [0.28, 2.20]
Van Dyck (CLARITY AD)	281.22	282.5	101	−5.95	271.83	97	8.6%	1.03 [0.73, 1.33]
**Subtotal (95% CI)**			**187**			**140**	**36.3%**	**1.27 [0.77, 1.77]**
Heterogeneity: $Tau^2 = 0.18$; $Chi^2 = 10.32$, df = 4 ($P = 0.04$); $I^2 = 61\%$								
Test for overall effect: Z = 5.01 (P < 0.00001)								
**Total (95% CI)**			**573**			**465**	**100.0%**	**1.12 [0.64, 1.61]**
Heterogeneity: $Tau^2 = 0.68$; $Chi^2 = 130.50$, df = 12 ($P < 0.00001$); $I^2 = 91\%$								
Test for overall effect: Z = 4.57 (P < 0.00001)								
Test for subgroup differences: $Chi^2 = 5.68$, df = 2 ($P = 0.06$), $I^2 = 64.8\%$								

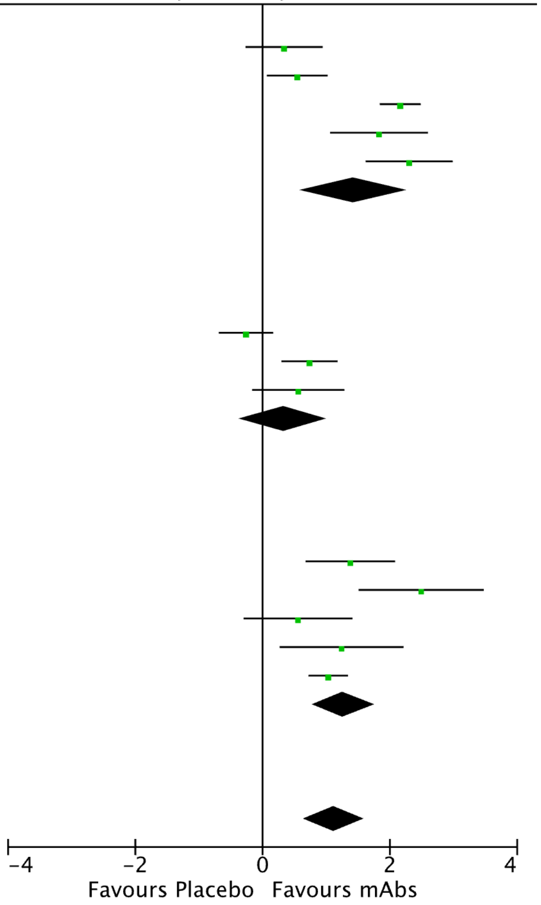

Figure S10. Forest plot of CSF $A\beta_{42}$ meta-analysis with subgroup analysis by monomer binding affinity

Study or Subgroup	mAbs Mean	mAbs SD	mAbs Total	Placebo Mean	Placebo SD	Placebo Total	Weight	Std. Mean Difference IV, Random, 95% CI
**1.5.1 Monomer Binding = Yes**								
Doody (EXPEDITION 1)	1,325.4	9,588.27	20	−1,902.1	11,526.61	25	14.0%	0.30 [−0.30, 0.89]
Doody (EXPEDITION 2)	2,156.8	11,080.1	44	−876.4	9,613.82	32	14.9%	0.29 [−0.17, 0.74]
Honig (EXPEDITION 3)	3,531.06	4,331.08	131	−993.4	3,833.71	126	16.0%	1.10 [0.84, 1.36]
Ostrowitzki (CREAD)	6,019.13	3,072.9	17	−99.19	3,339.18	22	12.6%	1.86 [1.09, 2.63]
Ostrowitzki (CREAD 2)	6,390.57	3,394.18	31	360	3,698.11	25	13.8%	1.68 [1.07, 2.30]
**Subtotal (95% CI)**			**243**			**230**	**71.2%**	**1.01 [0.46, 1.56]**
Heterogeneity: $Tau^2 = 0.32$; $Chi^2 = 24.77$, df = 4 ($P < 0.0001$); $I^2 = 84\%$								
Test for overall effect: Z = 3.58 (P = 0.0003)								
**1.5.2 Monomer Binding = Weak**								
Marguerite RoAD	−1,361.58	1,464.93	12	−1,258	2,520.32	23	13.1%	−0.05 [−0.74, 0.65]
**Subtotal (95% CI)**			**12**			**23**	**13.1%**	**−0.05 [−0.74, 0.65]**
Heterogeneity: Not applicable								
Test for overall effect: Z = 0.13 (P = 0.90)								
**1.5.3 Monomer Binding = No**								
Van Dyck (CLARITY AD)	−407.69	2,930.19	71	−93.64	2,844.33	71	15.7%	−0.11 [−0.44, 0.22]
**Subtotal (95% CI)**			**71**			**71**	**15.7%**	**−0.11 [−0.44, 0.22]**
Heterogeneity: Not applicable								
Test for overall effect: Z = 0.64 (P = 0.52)								
**Total (95% CI)**			**326**			**324**	**100.0%**	**0.70 [0.15, 1.26]**
Heterogeneity: $Tau^2 = 0.49$; $Chi^2 = 59.58$, df = 6 ($P < 0.00001$); $I^2 = 90\%$								
Test for overall effect: Z = 2.48 (P = 0.01)								
Test for subgroup differences: $Chi^2 = 11.97$, df = 2 (P = 0.003), $I^2 = 83.3\%$								

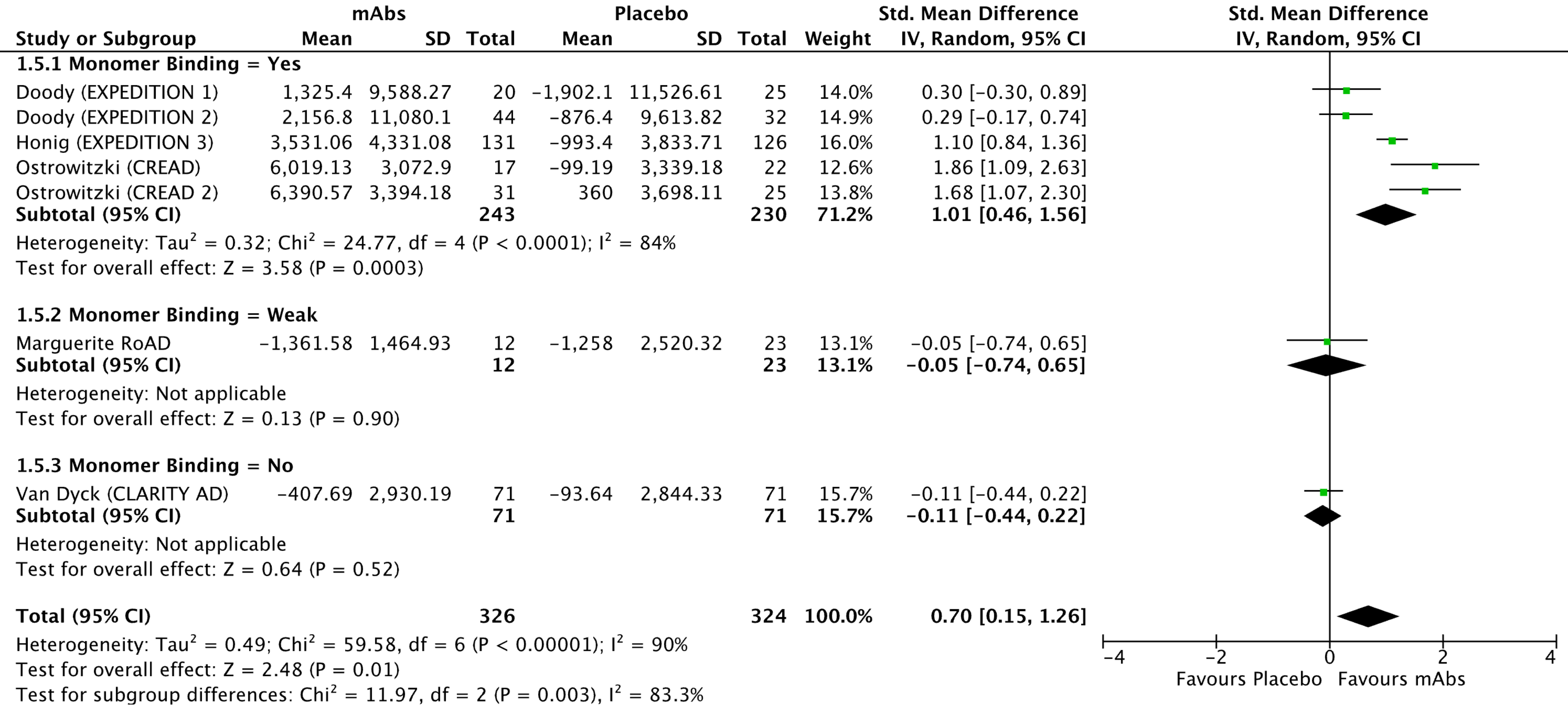

Figure S11. Forest plot of CSF $A\beta_{40}$ meta-analysis with subgroup analysis by monomer binding affinity

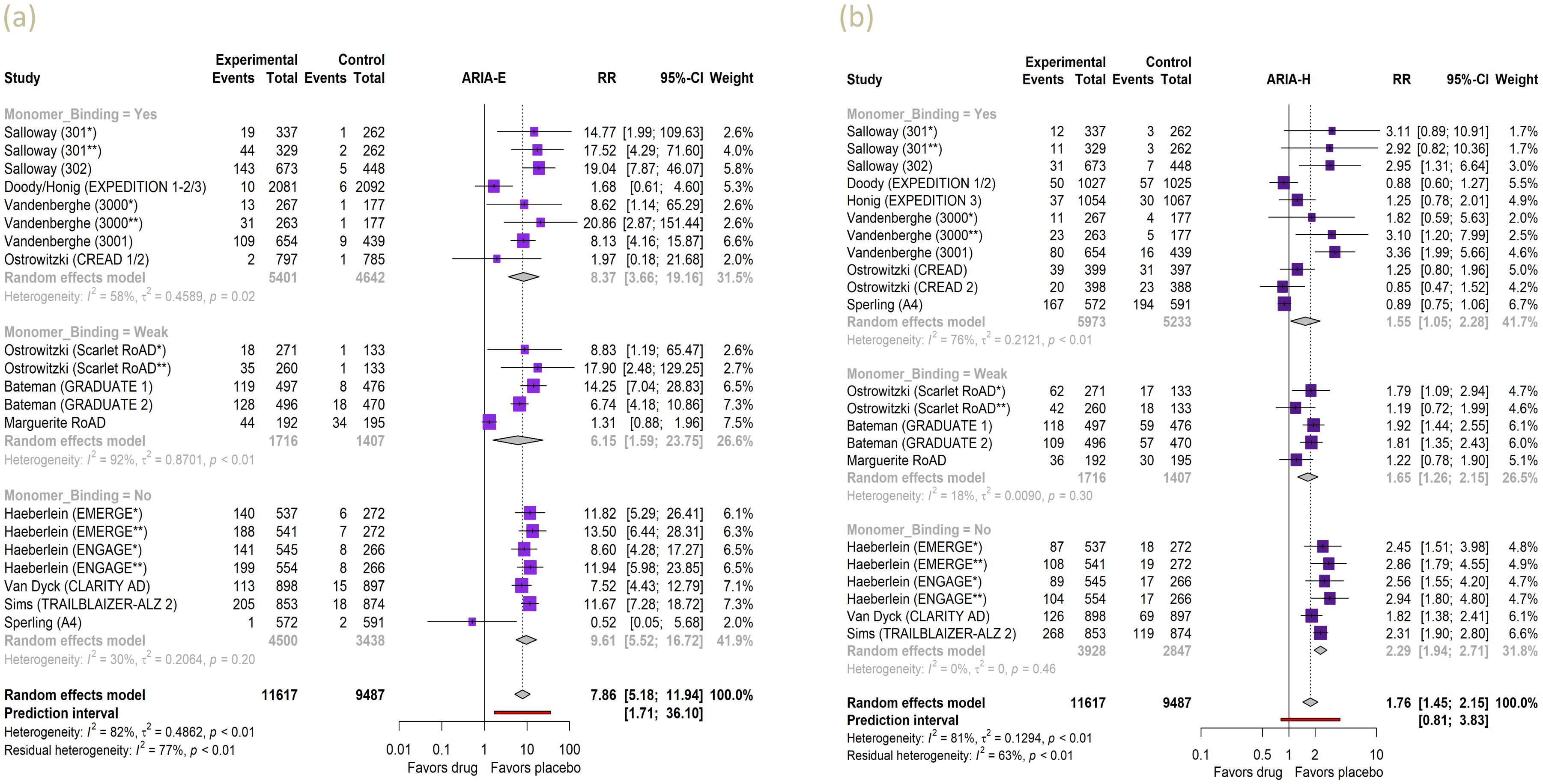

Figure S12. Forest plots of (a) Amyloid-Related Imaging Abnormalities with Edema/Effusion (ARIA-E) and (b) ARIA with microhemorrhages or superficial siderosis (ARIA-H) with subgroup analyses by binding affinity to Aβ monomers

Study or Subgroup	mAbs Events	mAbs Total	Placebo Events	Placebo Total	Weight	Risk Ratio M-H, Random, 95% CI
**1.2.1 Monomer Binding = Yes**						
Salloway (301*)	4	337	4	262	4.0%	0.78 [0.20, 3.08]
Salloway (301**)	7	329	3	262	4.2%	1.86 [0.49, 7.12]
Salloway (302)	15	673	5	448	6.7%	2.00 [0.73, 5.46]
Doody (EXPEDITION 1/2)	24	1027	19	1025	13.1%	1.26 [0.69, 2.29]
Honig (EXPEDITION 3)	9	1054	17	1067	9.2%	0.54 [0.24, 1.20]
Sperling (A4)	6	572	8	591	6.2%	0.77 [0.27, 2.22]
Vandenberghe (3000*)	1	267	2	172	1.5%	0.32 [0.03, 3.53]
Vandenberghe (3000**)	1	263	1	172	1.1%	0.65 [0.04, 10.39]
Vandenberghe (3001)	4	654	4	439	4.0%	0.67 [0.17, 2.67]
Ostrowitzki (CREAD)	8	404	5	405	5.8%	1.60 [0.53, 4.86]
Ostrowitzki (CREAD 2)	0	404	6	398	1.0%	0.08 [0.00, 1.34]
**Subtotal (95% CI)**		**5984**		**5241**	**56.9%**	**0.98 [0.68, 1.41]**
Total events	79		74			
Heterogeneity: $Tau^2 = 0.03$; $Chi^2 = 11.01$, df = 10 (P = 0.36); $I^2 = 9\%$						
Test for overall effect: Z = 0.10 (P = 0.92)						
**1.2.2 Monomer Binding = Weak**						
Ostrowitzki (Scarlet RoAD*)	0	271	3	133	1.0%	0.07 [0.00, 1.35]
Ostrowitzki (Scarlet RoAD**)	2	260	3	133	2.6%	0.34 [0.06, 2.02]
Bateman (GRADUATE 1)	3	503	10	481	4.5%	0.29 [0.08, 1.04]
Bateman (GRADUATE 2)	7	501	5	474	5.5%	1.32 [0.42, 4.14]
Marguerite RoAD	8	192	11	195	8.0%	0.74 [0.30, 1.80]
**Subtotal (95% CI)**		**1727**		**1416**	**21.6%**	**0.55 [0.26, 1.15]**
Total events	20		32			
Heterogeneity: $Tau^2 = 0.21$; $Chi^2 = 5.80$, df = 4 (P = 0.21); $I^2 = 31\%$						
Test for overall effect: Z = 1.60 (P = 0.11)						
**1.2.3 Monomer Binding = No**						
Haeberlein (EMERGE*)	0	544	3	274	1.0%	0.07 [0.00, 1.39]
Haeberlein (EMERGE**)	6	547	2	273	3.1%	1.50 [0.30, 7.37]
Haeberlein (ENGAGE*)	3	549	0	270	1.0%	3.45 [0.18, 66.53]
Haeberlein (ENGAGE**)	2	558	0	270	0.9%	2.42 [0.12, 50.31]
Sims (TRAILBLAZER-ALZ 2)	16	853	10	874	9.5%	1.64 [0.75, 3.59]
Van Dyck (CLARITY AD)	6	898	7	897	5.9%	0.86 [0.29, 2.54]
**Subtotal (95% CI)**		**3949**		**2858**	**21.5%**	**1.26 [0.70, 2.27]**
Total events	33		22			
Heterogeneity: $Tau^2 = 0.03$; $Chi^2 = 5.23$, df = 5 (P = 0.39); $I^2 = 4\%$						
Test for overall effect: Z = 0.76 (P = 0.45)						
**Total (95% CI)**		**11660**		**9515**	**100.0%**	**0.91 [0.68, 1.23]**
Total events	132		128			
Heterogeneity: $Tau^2 = 0.09$; $Chi^2 = 25.64$, df = 21 (P = 0.22); $I^2 = 18\%$						
Test for overall effect: Z = 0.61 (P = 0.55)						
Test for subgroup differences: $Chi^2 = 3.03$, df = 2 (P = 0.22), $I^2 = 33.9\%$						

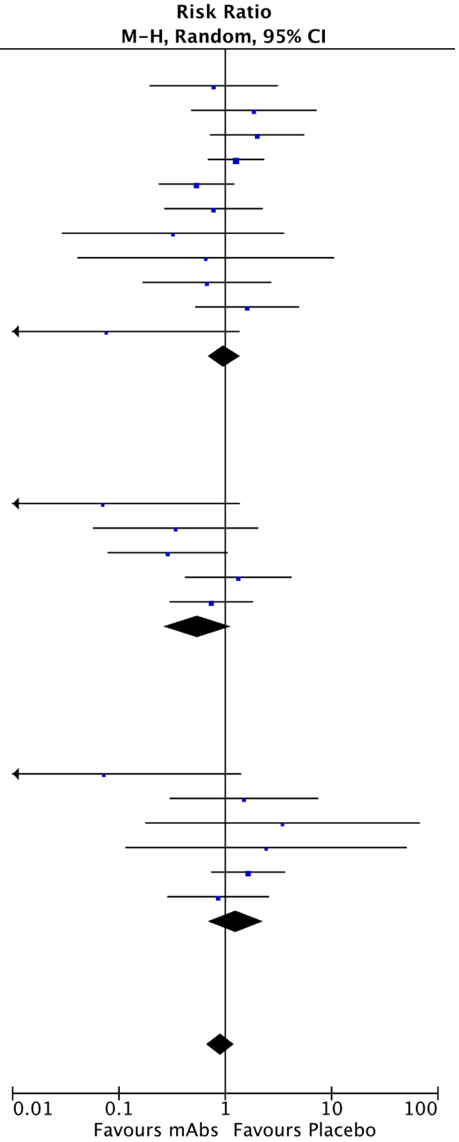

Figure S13. Forest plot of all-cause mortality meta-analysis with subgroup analysis by monomer binding affinity

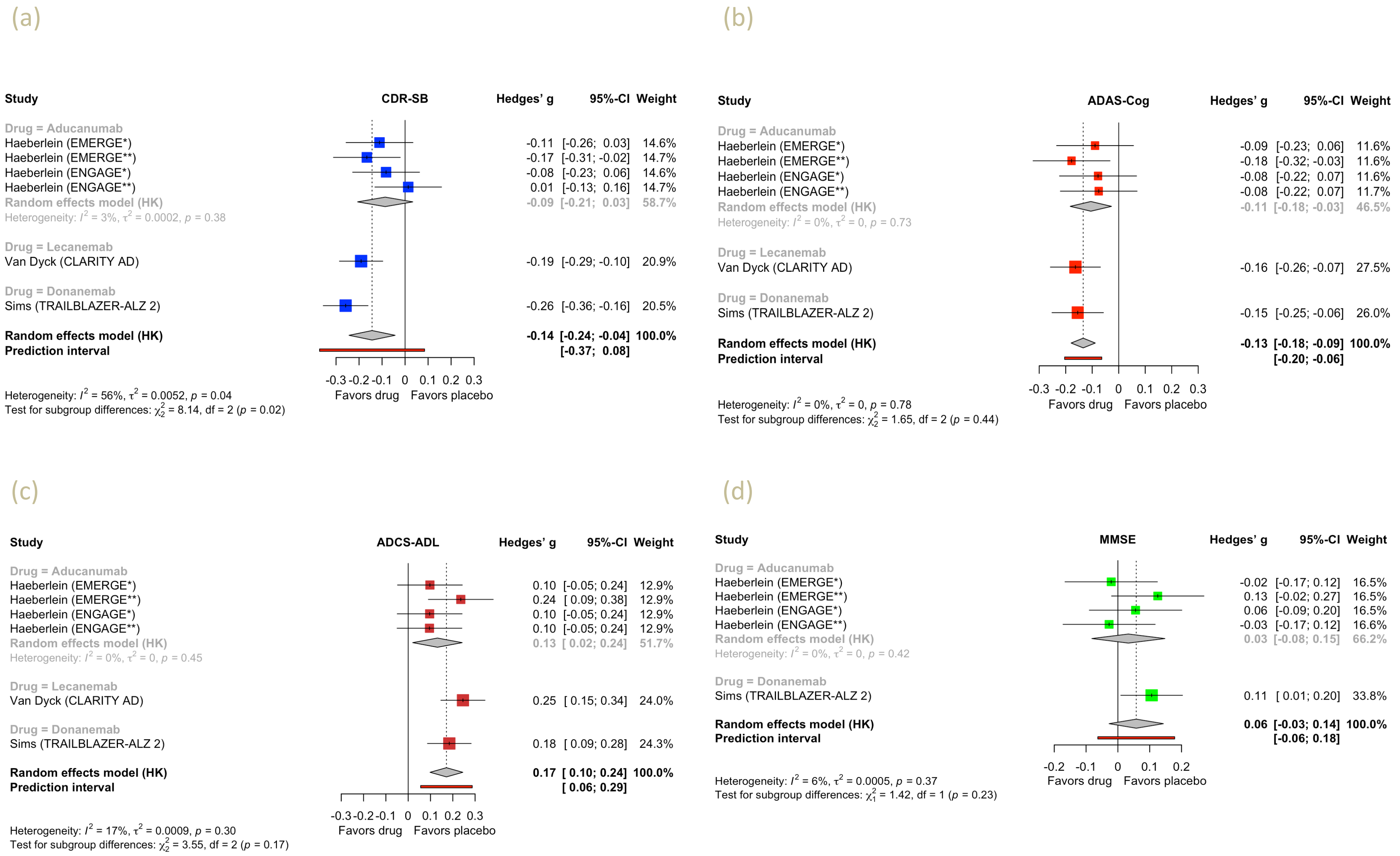

Figure S14. Forest plots of (a) Clinical Dementia Rating Scale Sum of Boxes (CDR-SB), (b) AD Assessment Scale–Cognitive Subscale (ADAS-Cog), (c) AD Cooperative Study – Activities of Daily Living (ADCS-ADL), (d) Mini Mental Examination State (MMSE) meta-analyses of FDA-approved monoclonal antibodies only with subgroup analyses by drug

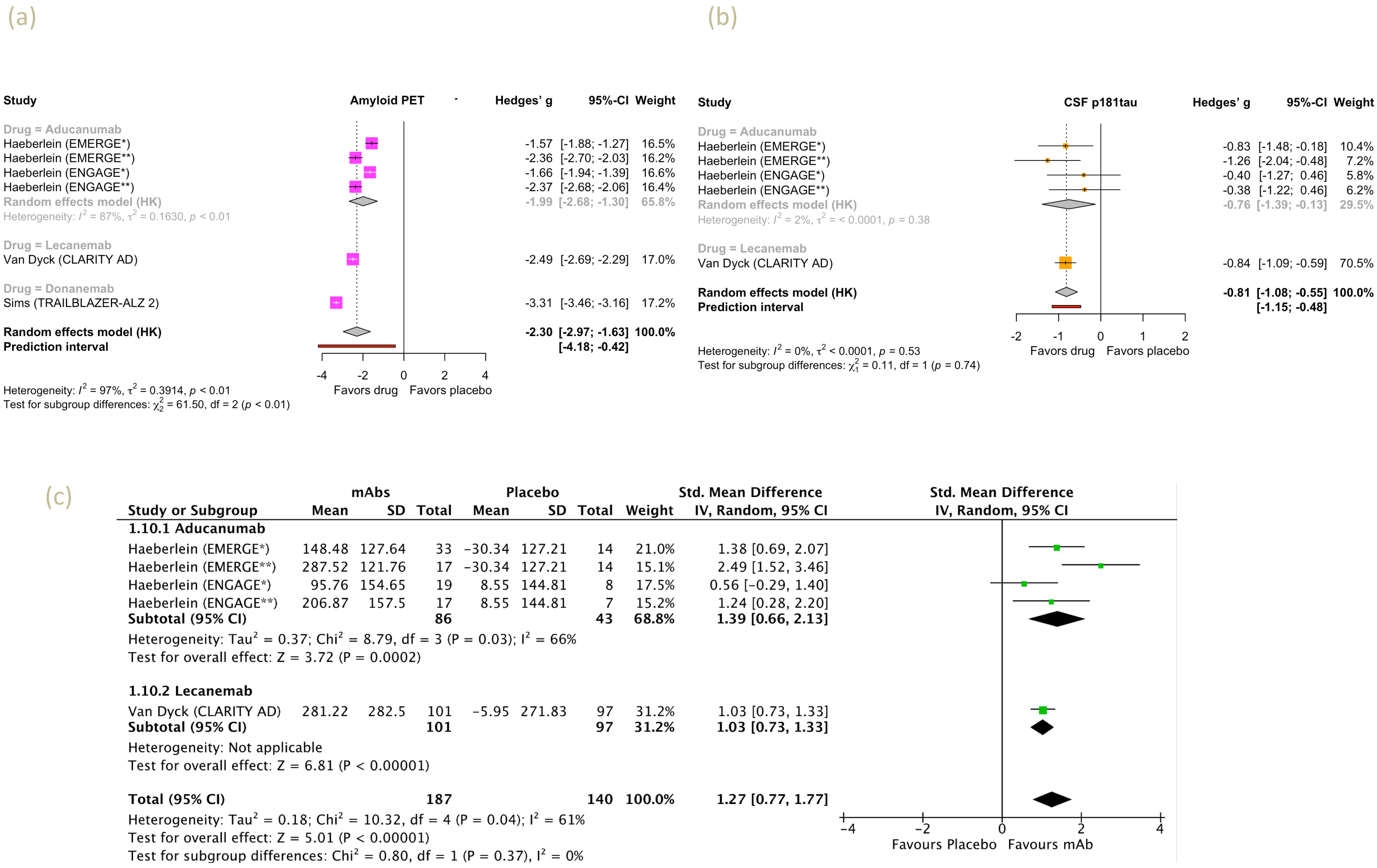

Figure S15. Forest plots of (a) Amyloid PET, (b) CSF p181-tau, (c) CSF Aβ_{42} meta-analyses of FDA-approved monoclonal antibodies only with subgroup analyses by drug

(a)

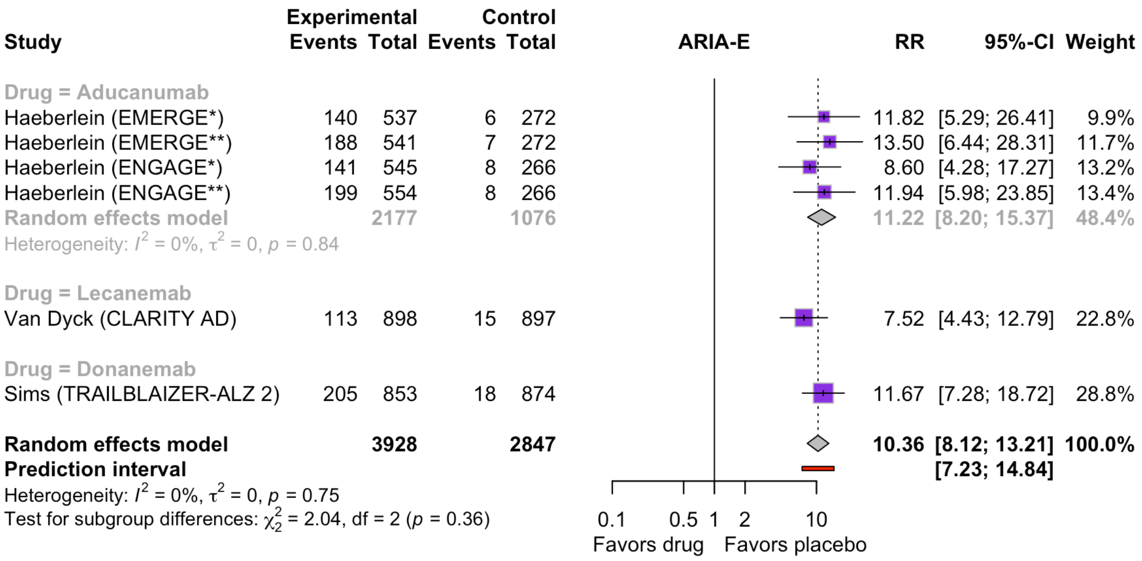

(b)

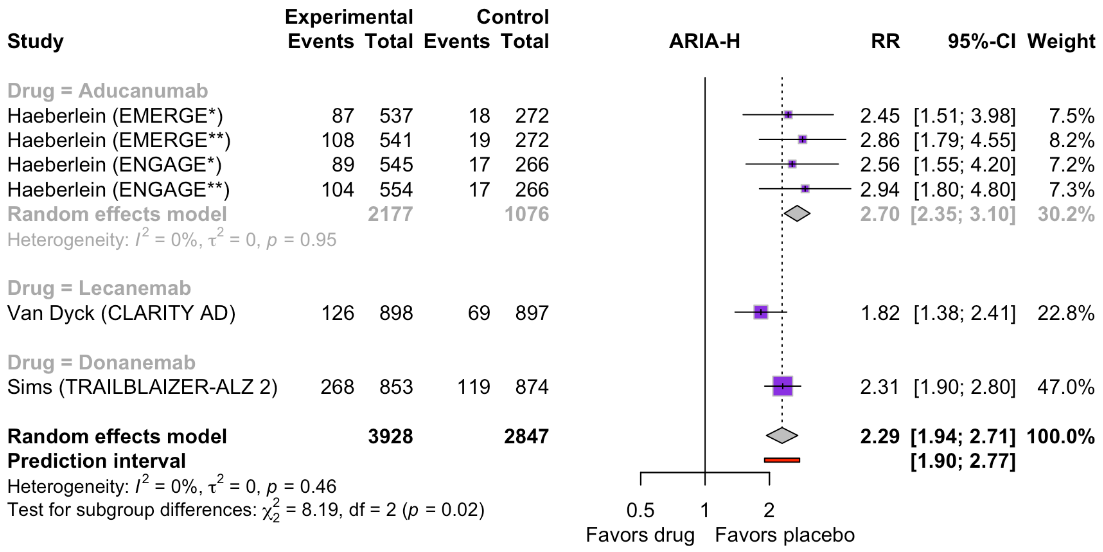

Figure S16. Forest plots of (a) Amyloid-Related Imaging Abnormalities with Edema/Effusion (ARIA-E) and (b) ARIA with microhemorrhages or superficial siderosis (ARIA-H) meta-analysis of FDA-approved antibodies only with subgroup analyses by drug

Study or Subgroup	mAbs Events	mAbs Total	Placebo Events	Placebo Total	Weight	Risk Ratio M-H, Random, 95% CI
**1.11.1 Aducanumab**						
Haeberlein (EMERGE*)	0	544	3	274	3.9%	0.07 [0.00, 1.39]
Haeberlein (EMERGE**)	6	547	2	273	13.2%	1.50 [0.30, 7.37]
Haeberlein (ENGAGE*)	3	549	0	270	3.9%	3.45 [0.18, 66.53]
Haeberlein (ENGAGE**)	2	558	0	270	3.7%	2.42 [0.12, 50.31]
**Subtotal (95% CI)**		**2198**		**1087**	**24.8%**	**1.07 [0.24, 4.80]**
Total events	11		5			
Heterogeneity: $Tau^2 = 0.74$; $Chi^2 = 4.32$, df = 3 (P = 0.23); $I^2 = 31\%$						
Test for overall effect: Z = 0.08 (P = 0.93)						
**1.11.2 Lecanemab**						
Van Dyck (CLARITY AD)	6	898	7	897	27.0%	0.86 [0.29, 2.54]
**Subtotal (95% CI)**		**898**		**897**	**27.0%**	**0.86 [0.29, 2.54]**
Total events	6		7			
Heterogeneity: Not applicable						
Test for overall effect: Z = 0.28 (P = 0.78)						
**1.11.3 Donanemab**						
Sims (TRAILBLAZER-ALZ 2)	16	853	10	874	48.2%	1.64 [0.75, 3.59]
**Subtotal (95% CI)**		**853**		**874**	**48.2%**	**1.64 [0.75, 3.59]**
Total events	16		10			
Heterogeneity: Not applicable						
Test for overall effect: Z = 1.24 (P = 0.22)						
**Total (95% CI)**		**3949**		**2858**	**100.0%**	**1.26 [0.70, 2.27]**
Total events	33		22			
Heterogeneity: $Tau^2 = 0.03$; $Chi^2 = 5.23$, df = 5 (P = 0.39); $I^2 = 4\%$						
Test for overall effect: Z = 0.76 (P = 0.45)						
Test for subgroup differences: $Chi^2 = 0.96$, df = 2 (P = 0.62), $I^2 = 0\%$						

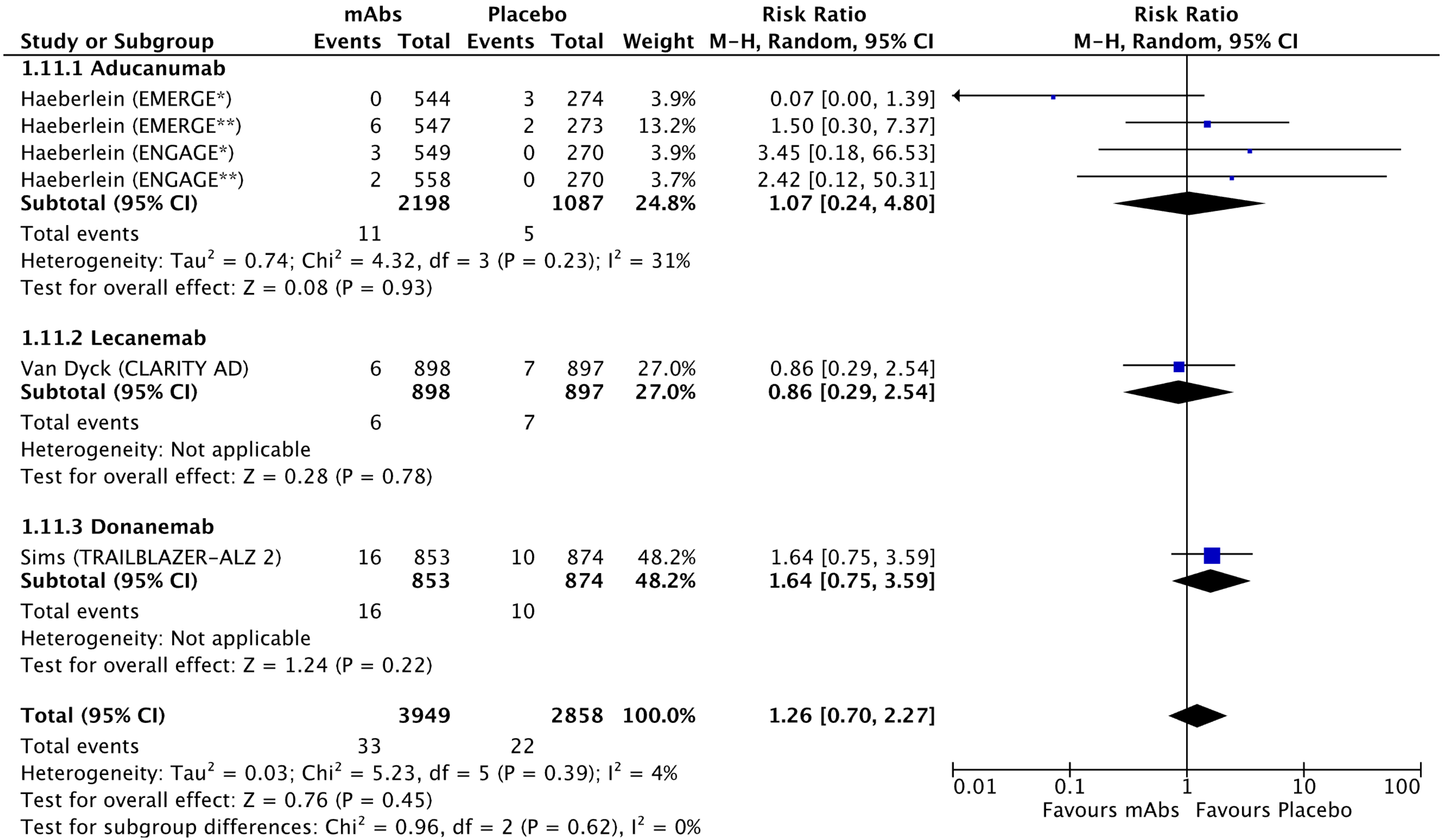

Figure S17. Forest plots of all-cause mortality meta-analysis of FDA-approved antibodies only with subgroup analyses by drug
